# Supplementary material for: Nuts and Cardio-Metabolic Disease: A Review of Meta-Analyses
Source: Nutrients. 2018 Dec 6;10(12):1935. doi: 10.3390/nu10121935 (PMC6316378; doi:10.3390/nu10121935)

Supplementary Materials

Supplementary Figure 1. Total nuts and all-cause mortality, high *vs.* low analysis

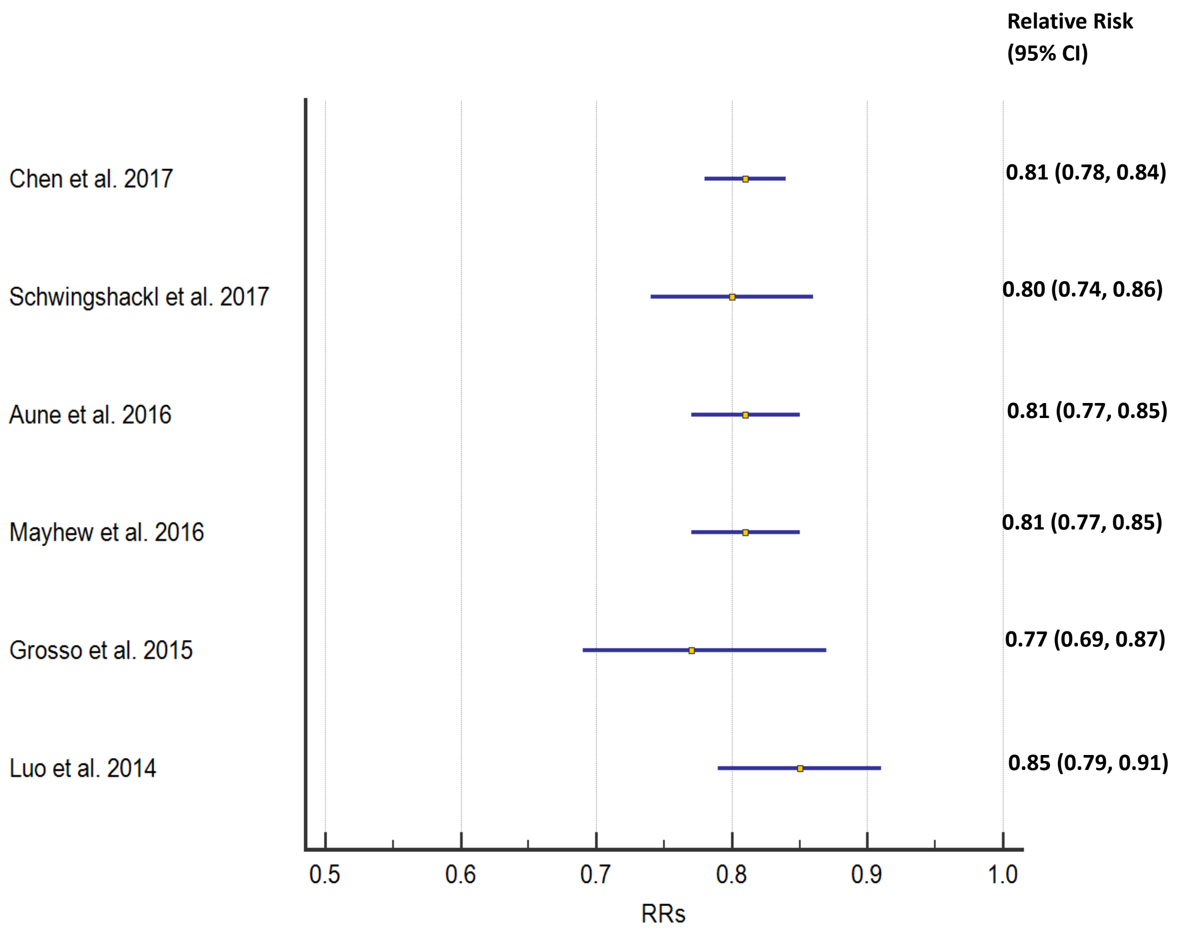

Supplementary Figure 2. Total nuts and all-cause mortality, dose-response analysis

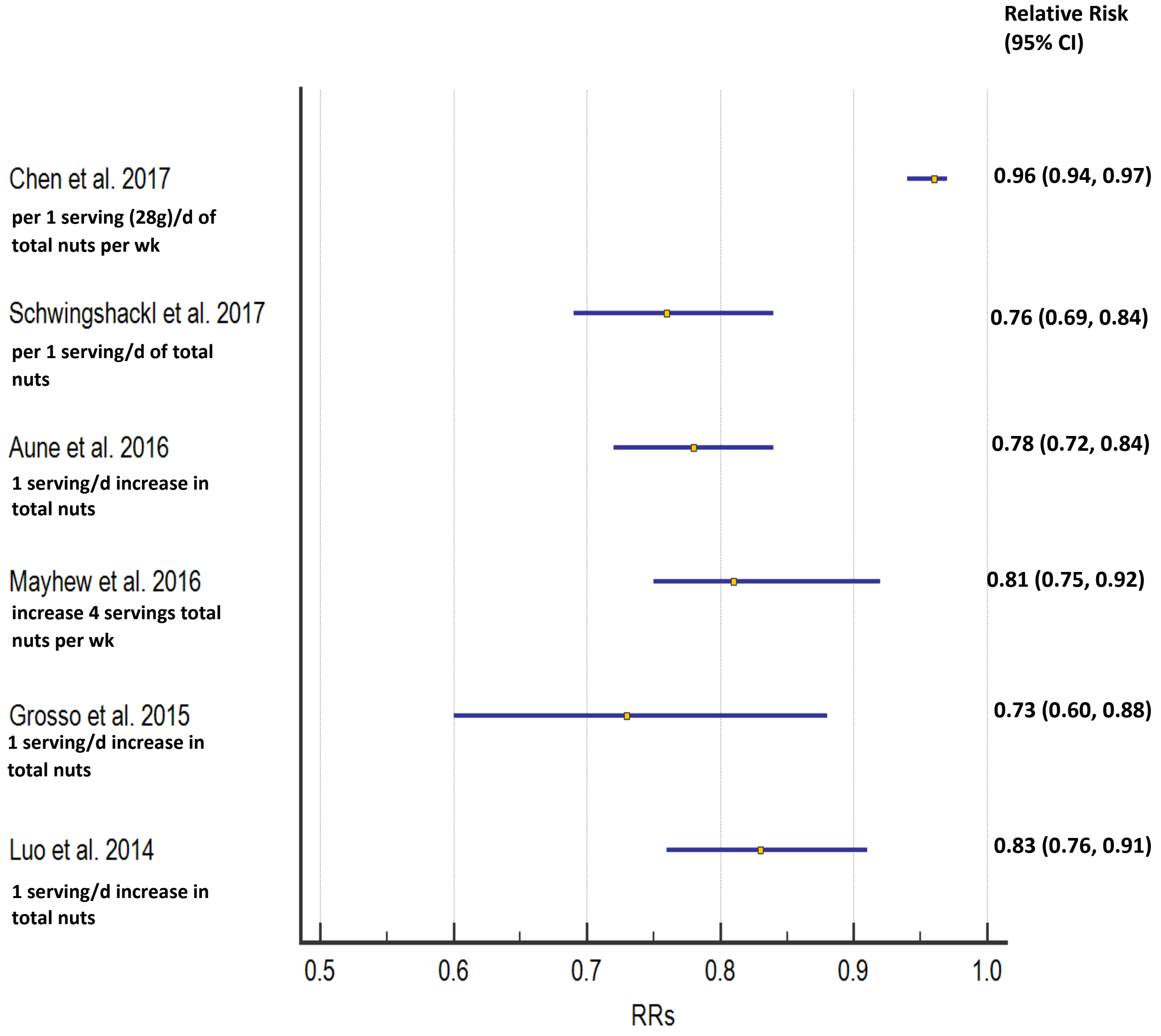

Supplementary Figure 3. Peanuts and all-cause mortality, high *vs.* low analysis

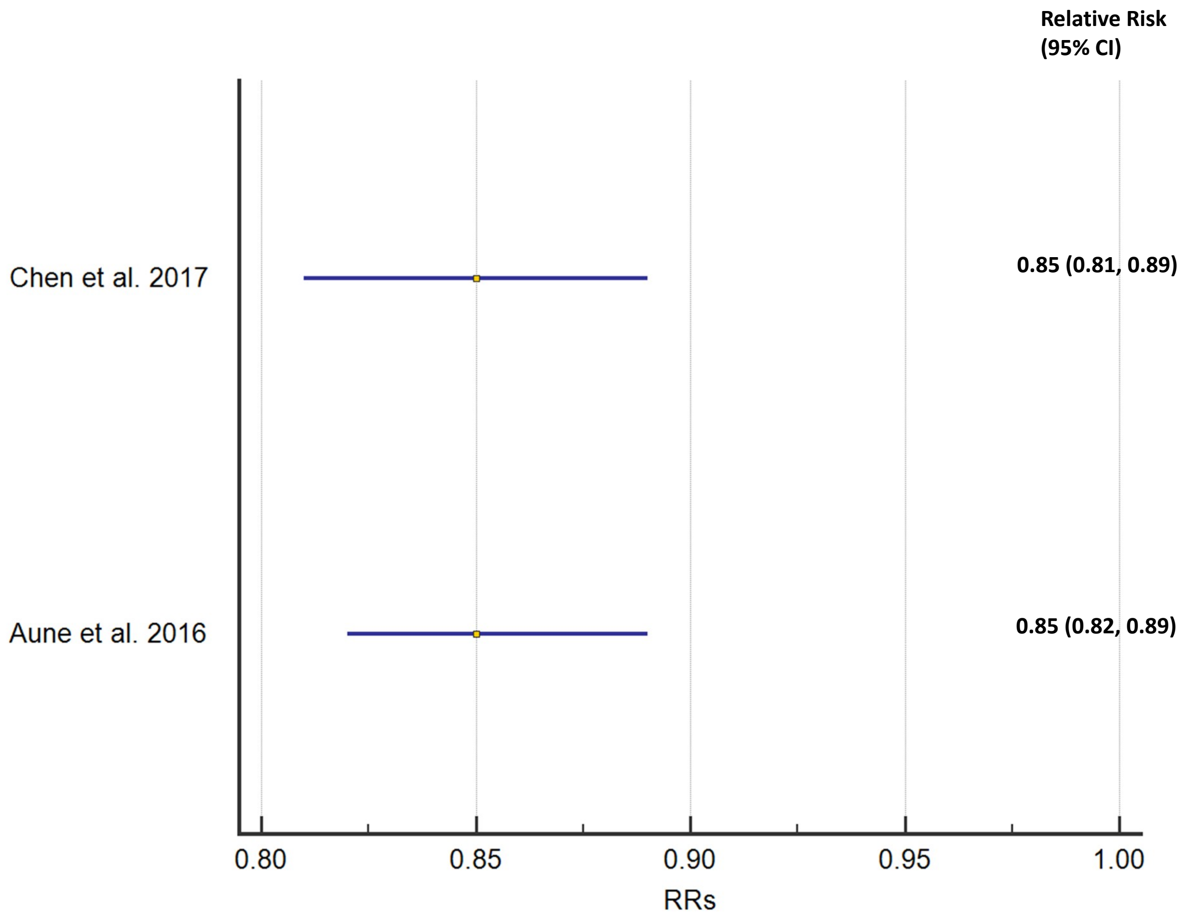

Supplementary Figure 4. Tree nuts and all-cause mortality, high *vs.* low analysis

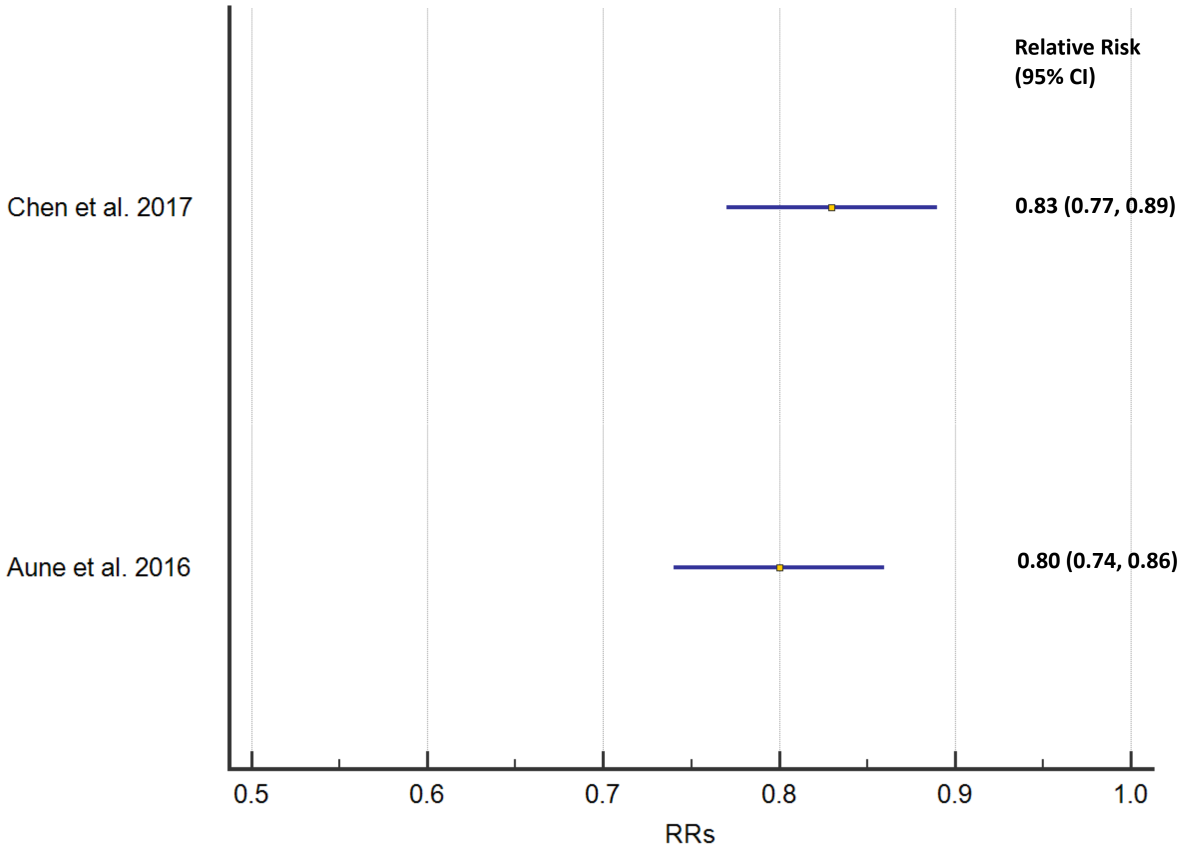

Supplementary Figure 5. Total nuts and coronary heart disease mortality, high *vs.* low analysis

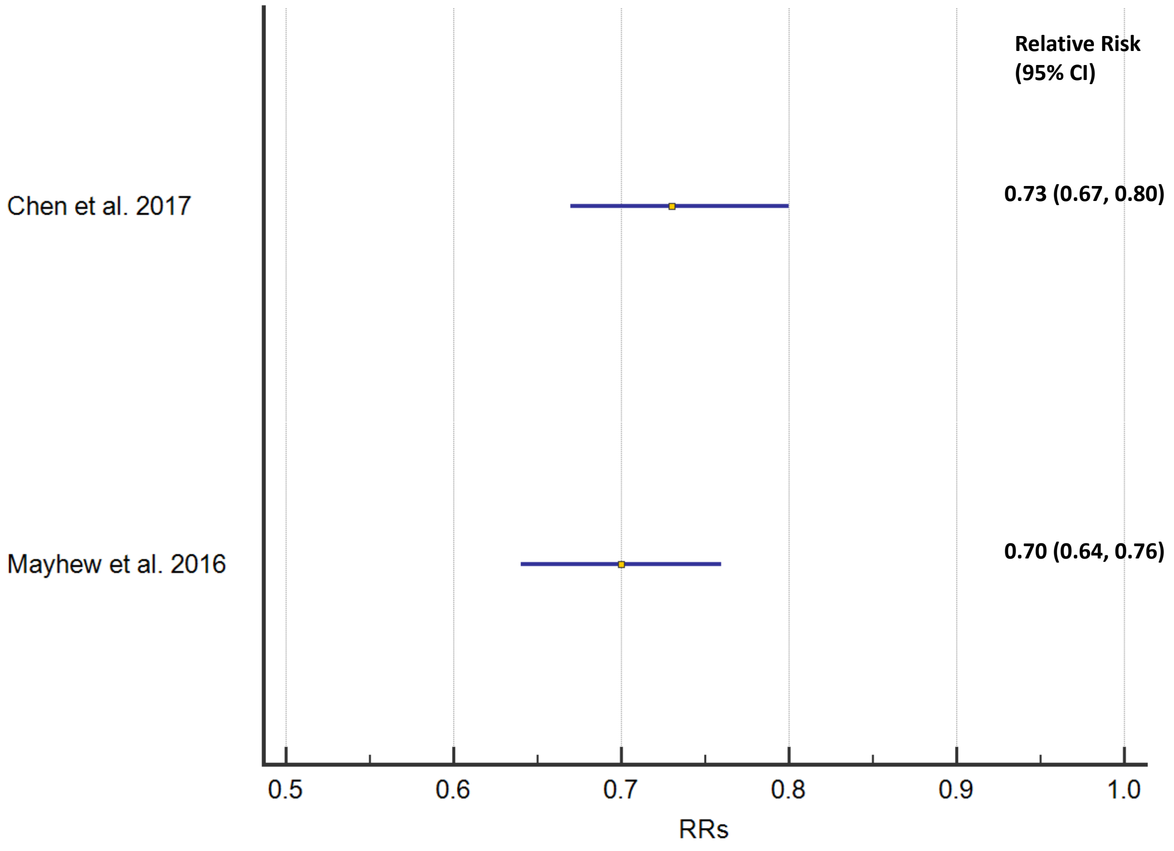

Supplementary Figure 6. Total nuts and coronary heart disease mortality, dose-response analysis

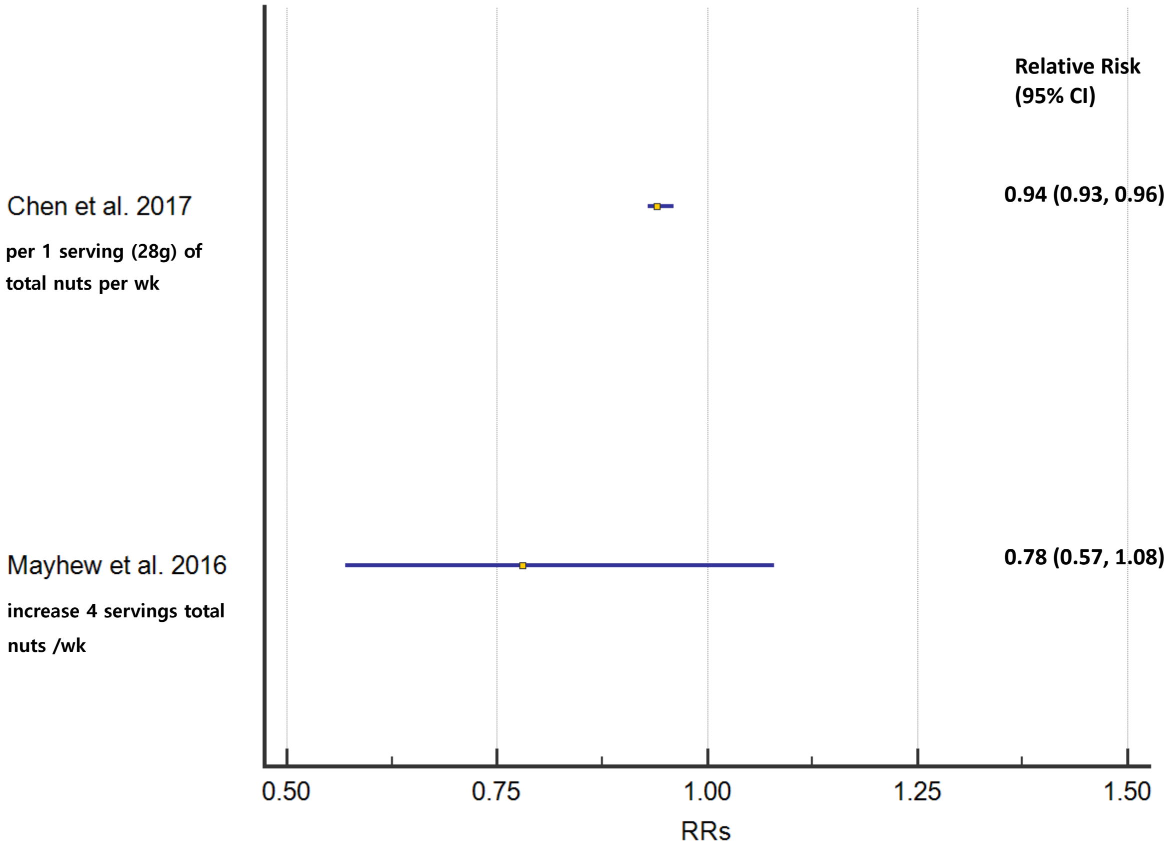

Supplementary Figure 7. Total nuts and cardiovascular disease mortality, high *vs.* low analysis

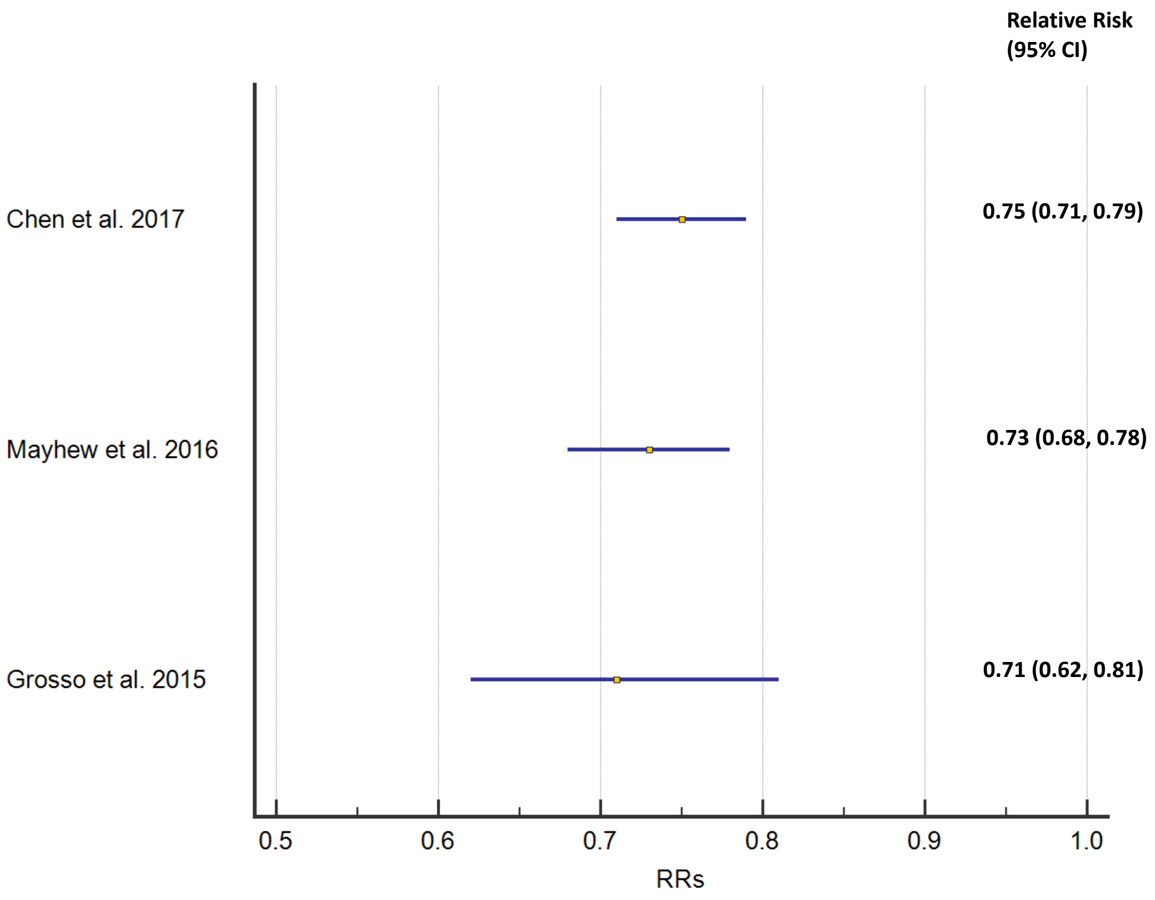

Supplementary Figure 8. Total nuts and cardiovascular disease mortality, dose-response analysis

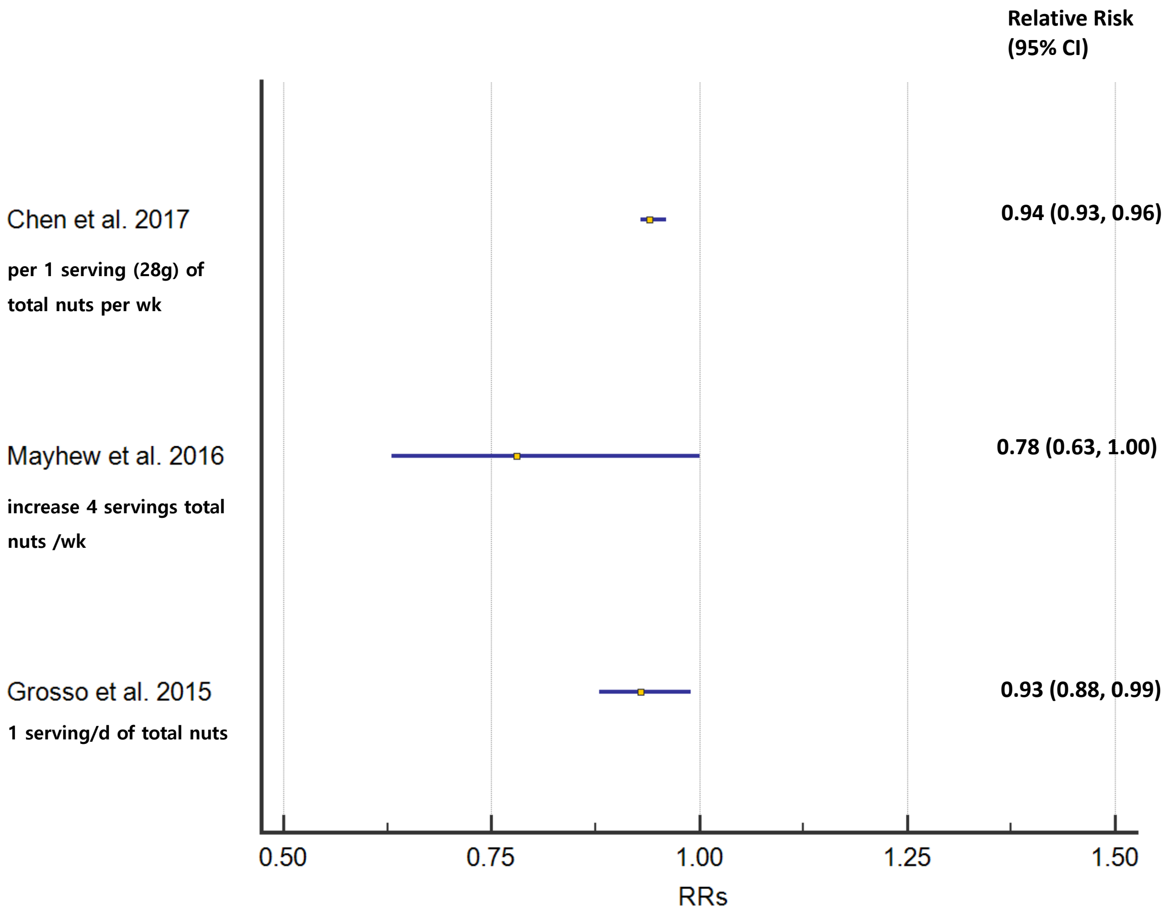

Supplementary Figure 9. Total nuts and stroke mortality, high *vs.* low analysis

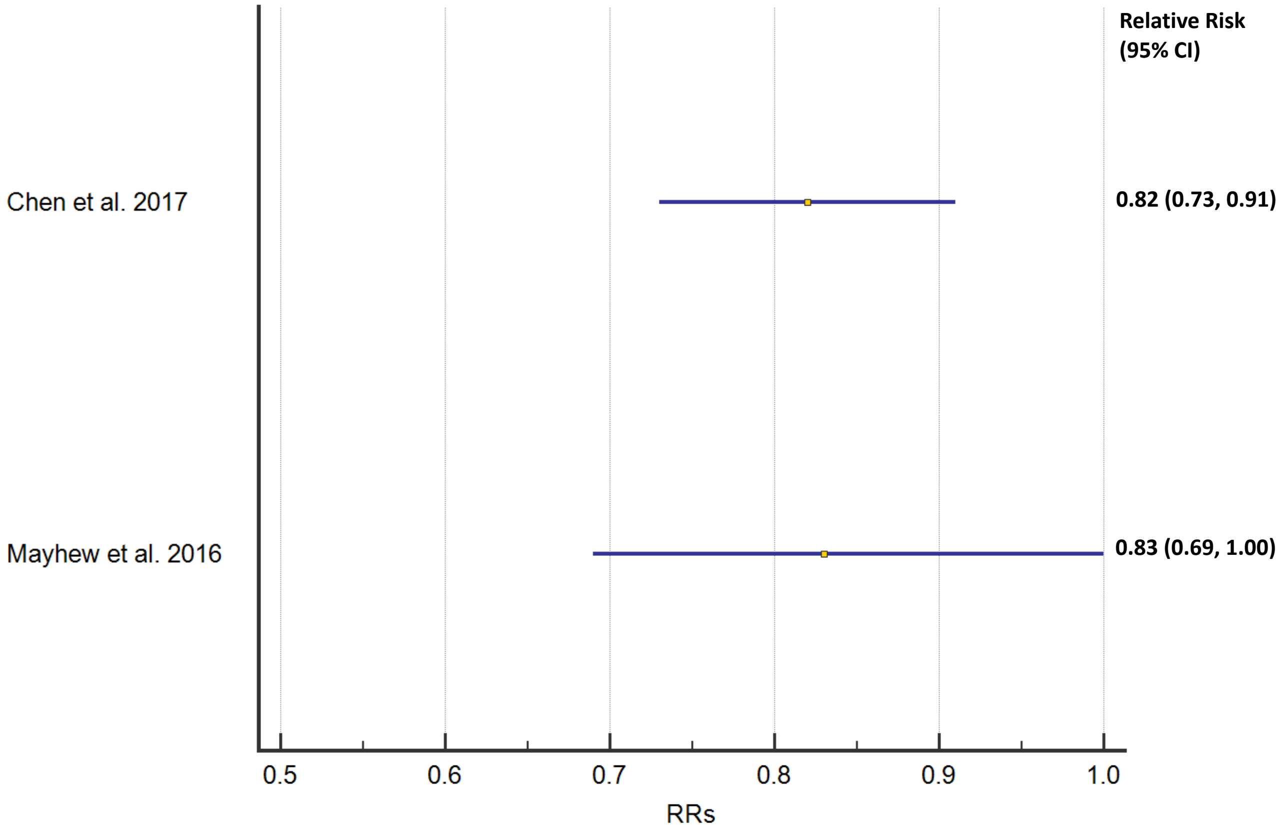

Supplementary Figure 10. Total nuts and stroke mortality, dose-response analysis

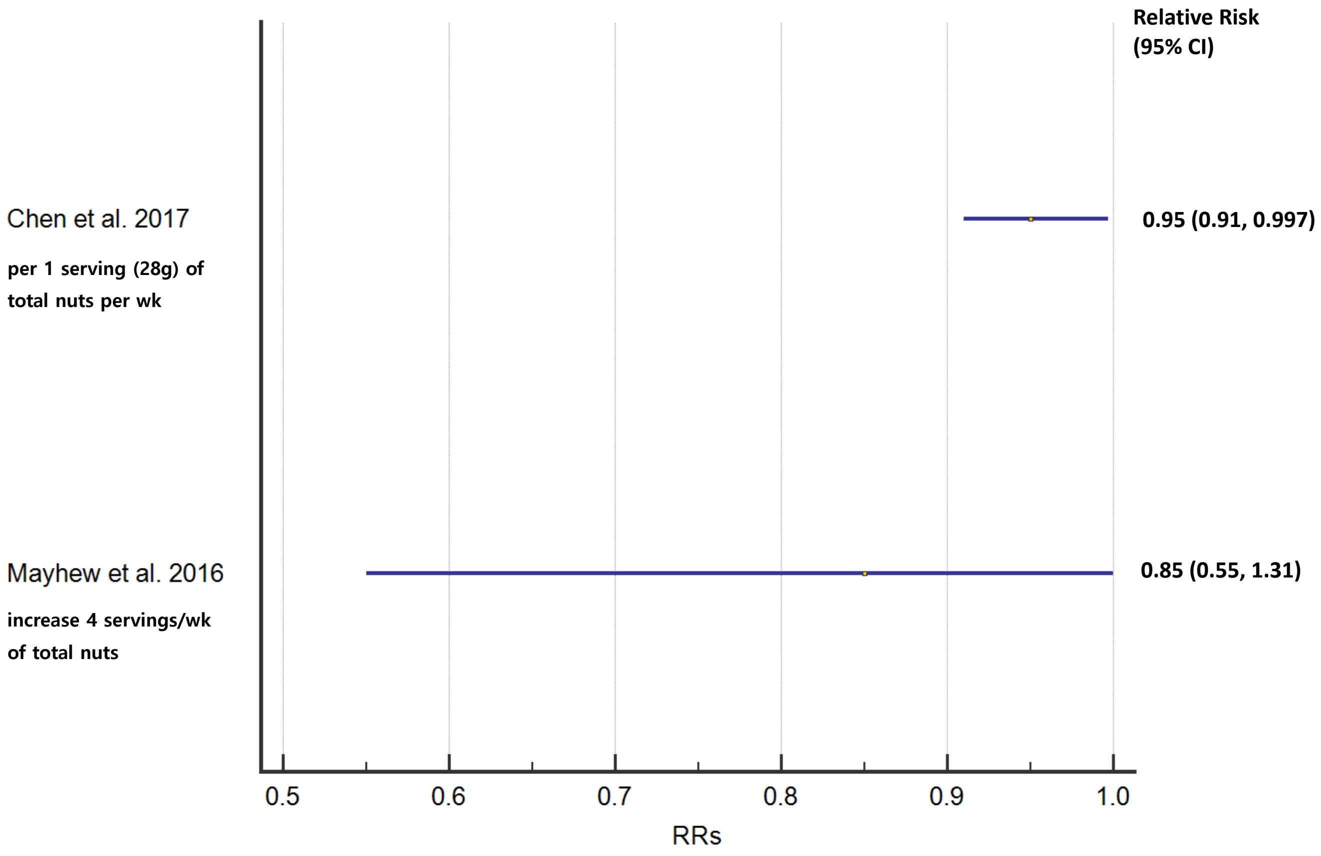

Supplementary Figure 11. Total nuts and type 2 diabetes, high *vs.* low analysis

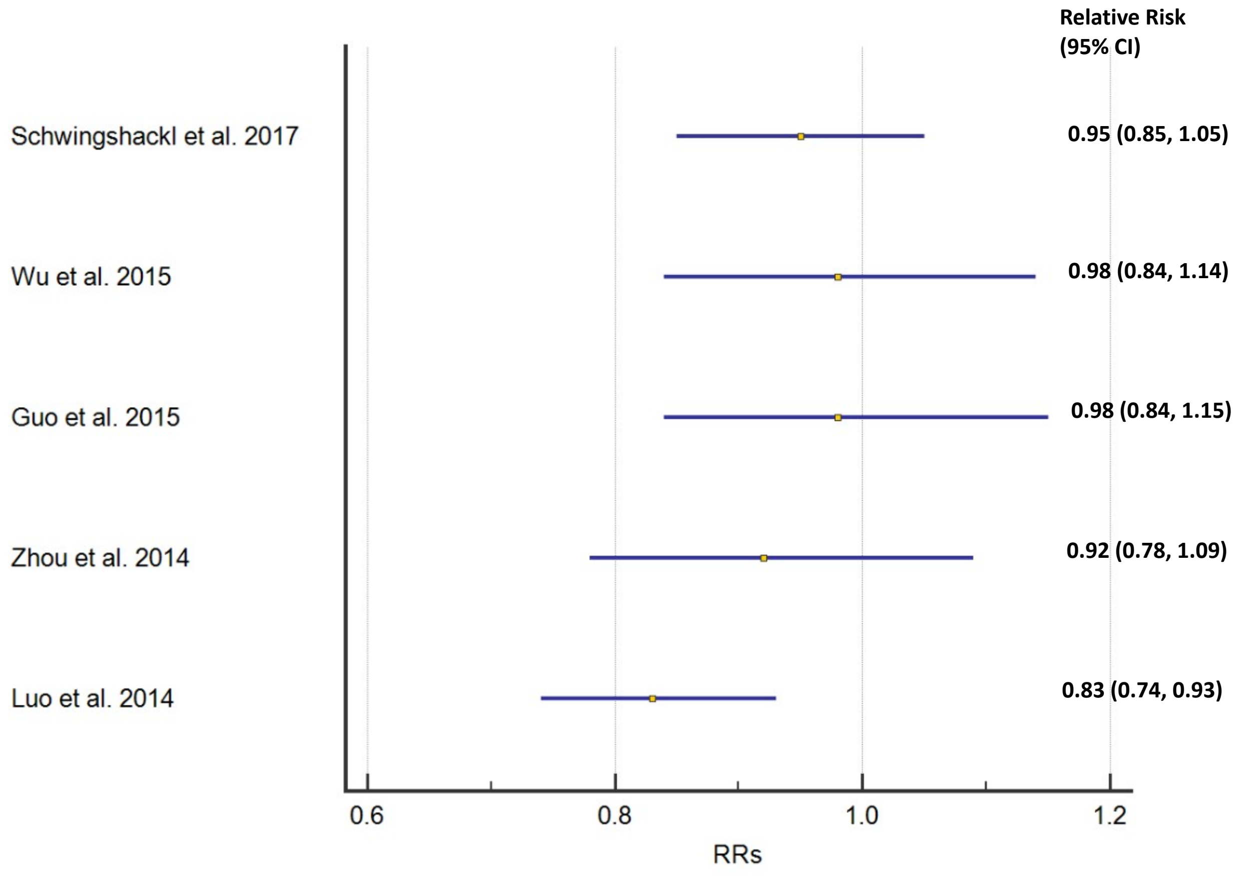

Supplementary Figure 12. Total nuts and type 2 diabetes, dose-response analysis

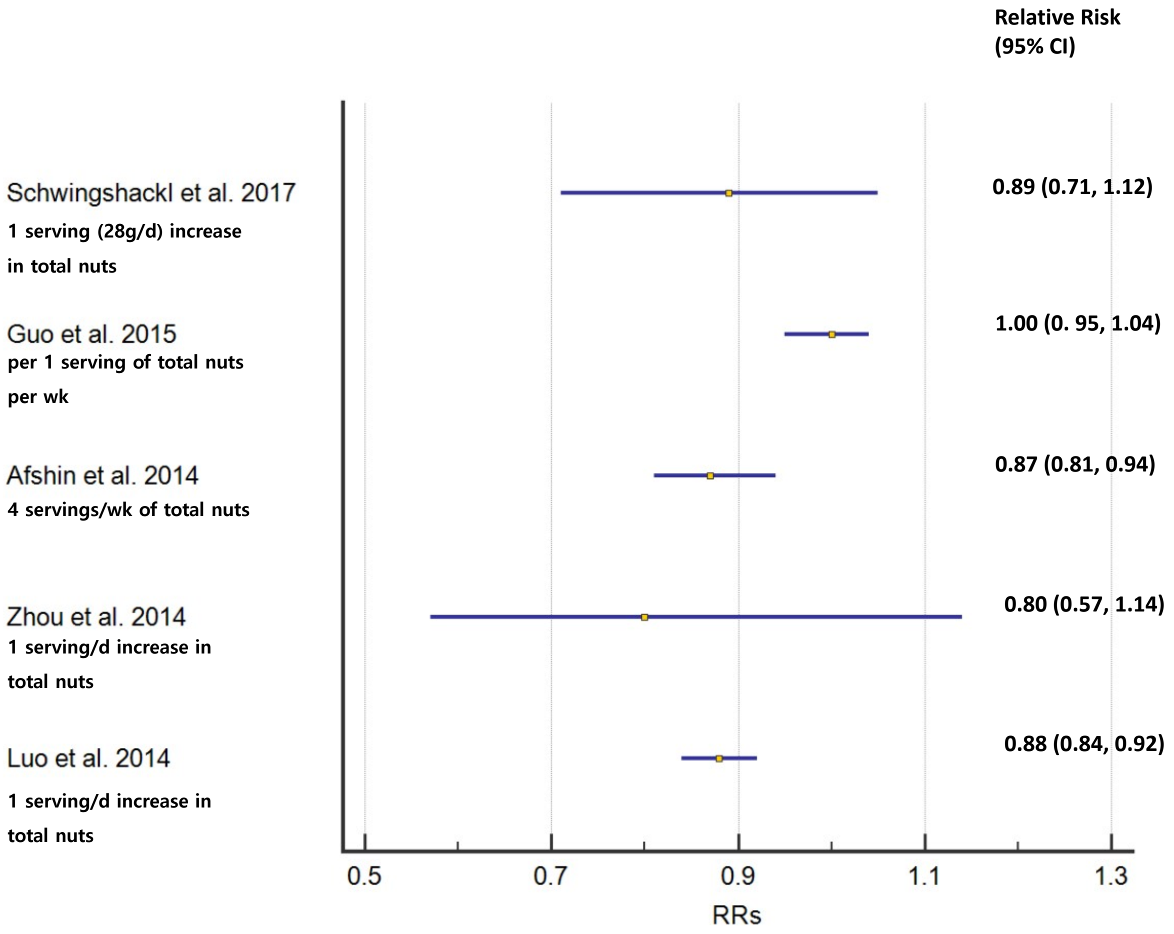

Supplementary Figure 13. Total nuts and total cardiovascular disease, high *vs.* low analysis

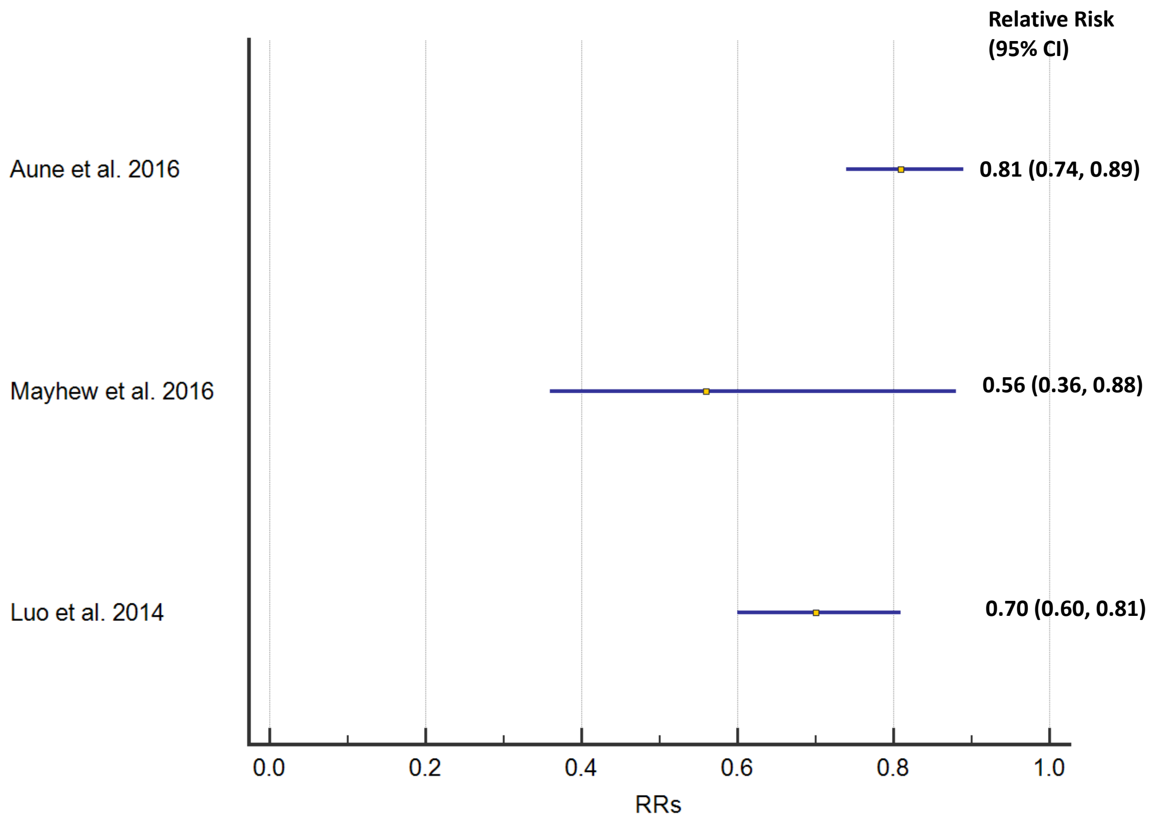

Supplementary Figure 14. Total nuts and total cardiovascular disease, dose-response analysis

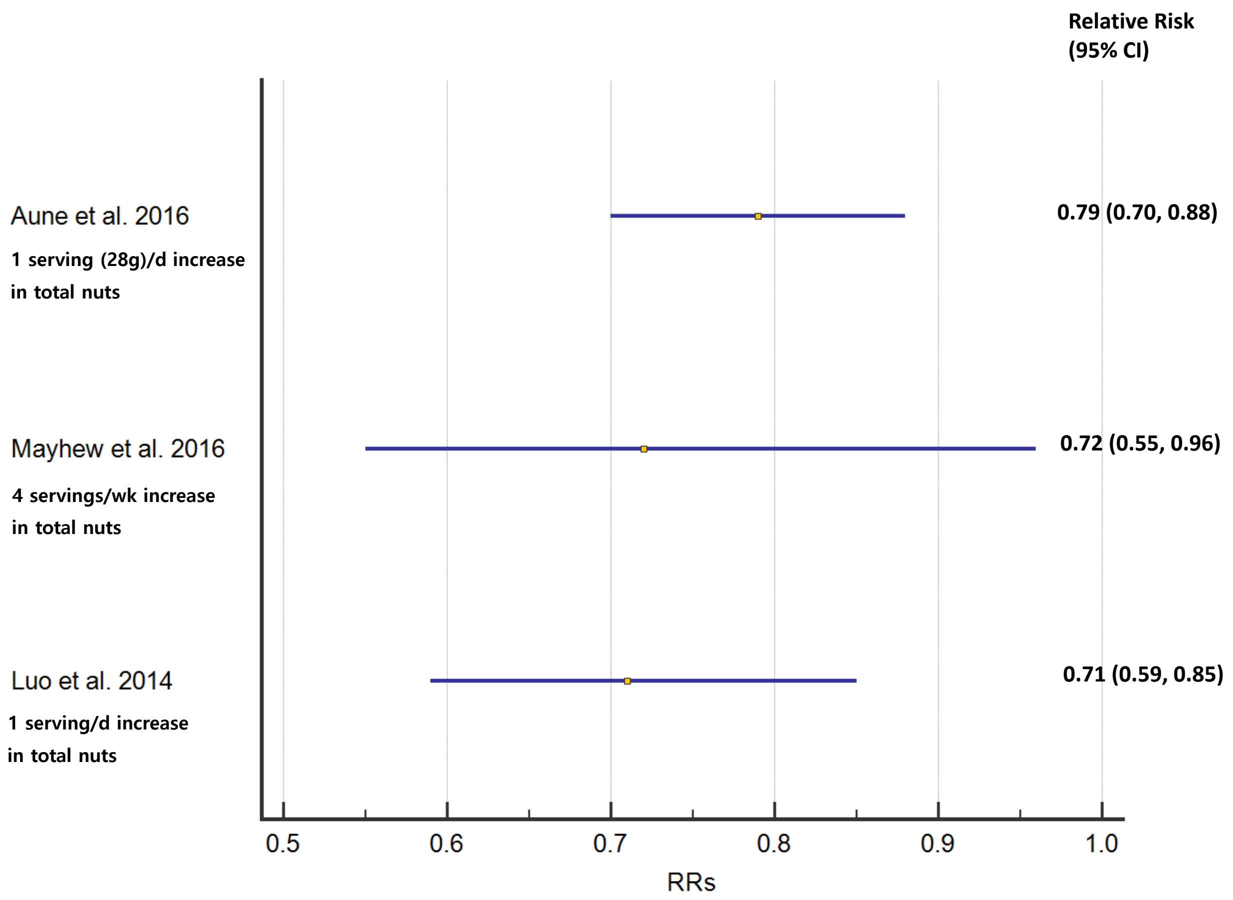

Supplementary Figure 15. Total nuts and hypertension, high *vs.* low analysis

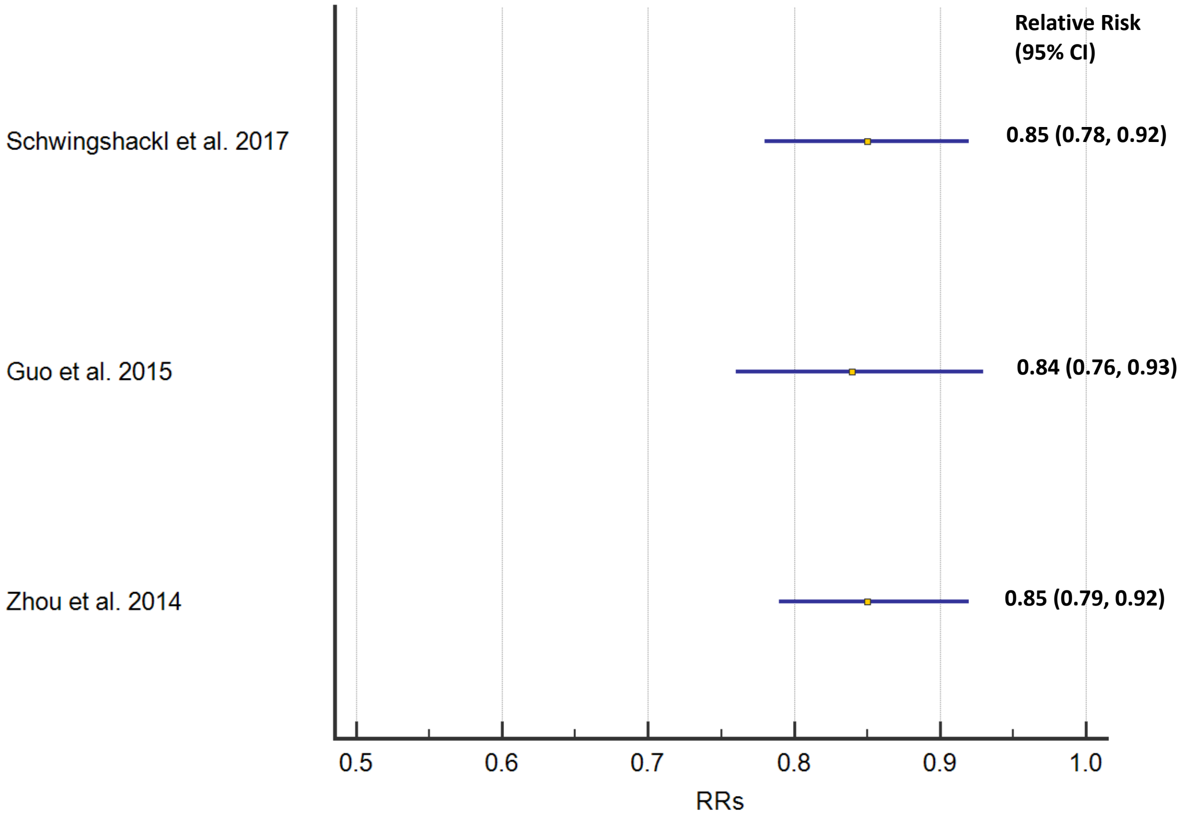

Supplementary Figure 16. Total nuts and hypertension, dose-response analysis

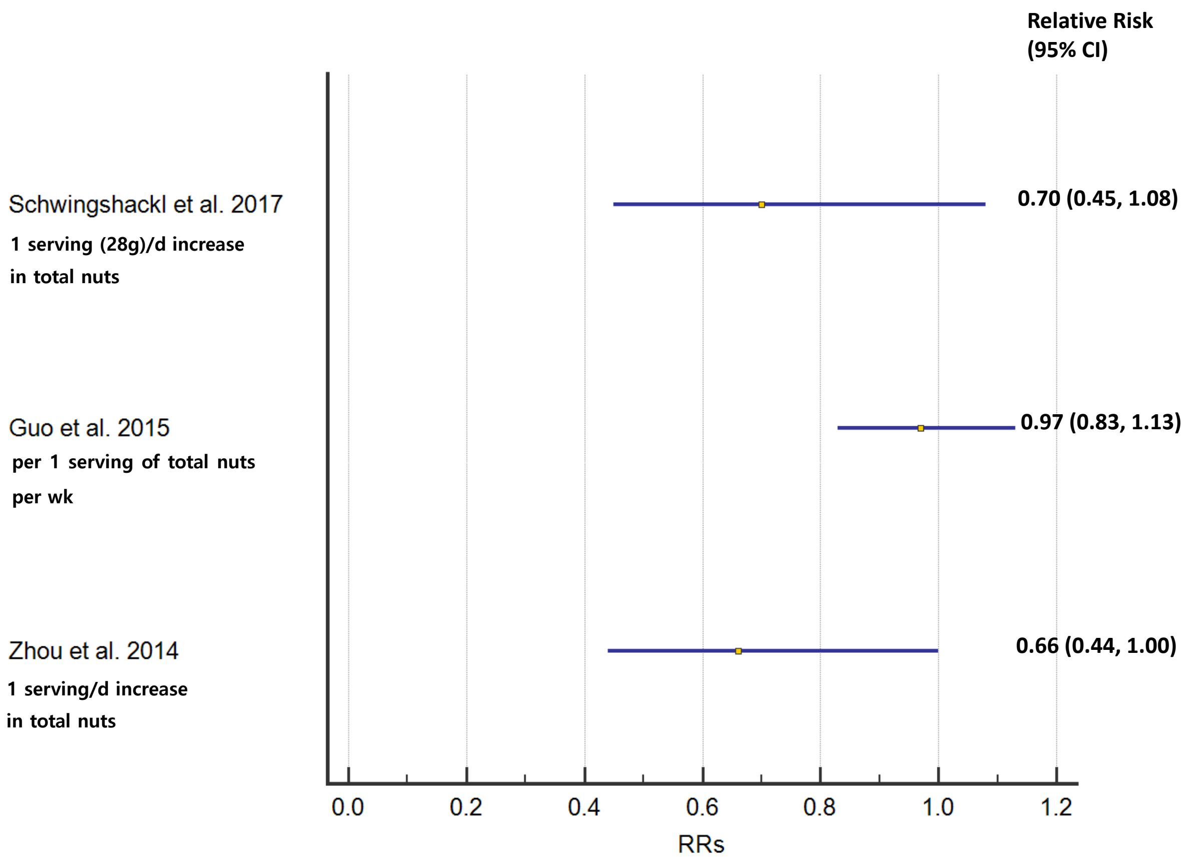

Supplementary Figure 17. Total nuts and total coronary heart disease, high *vs.* low analysis

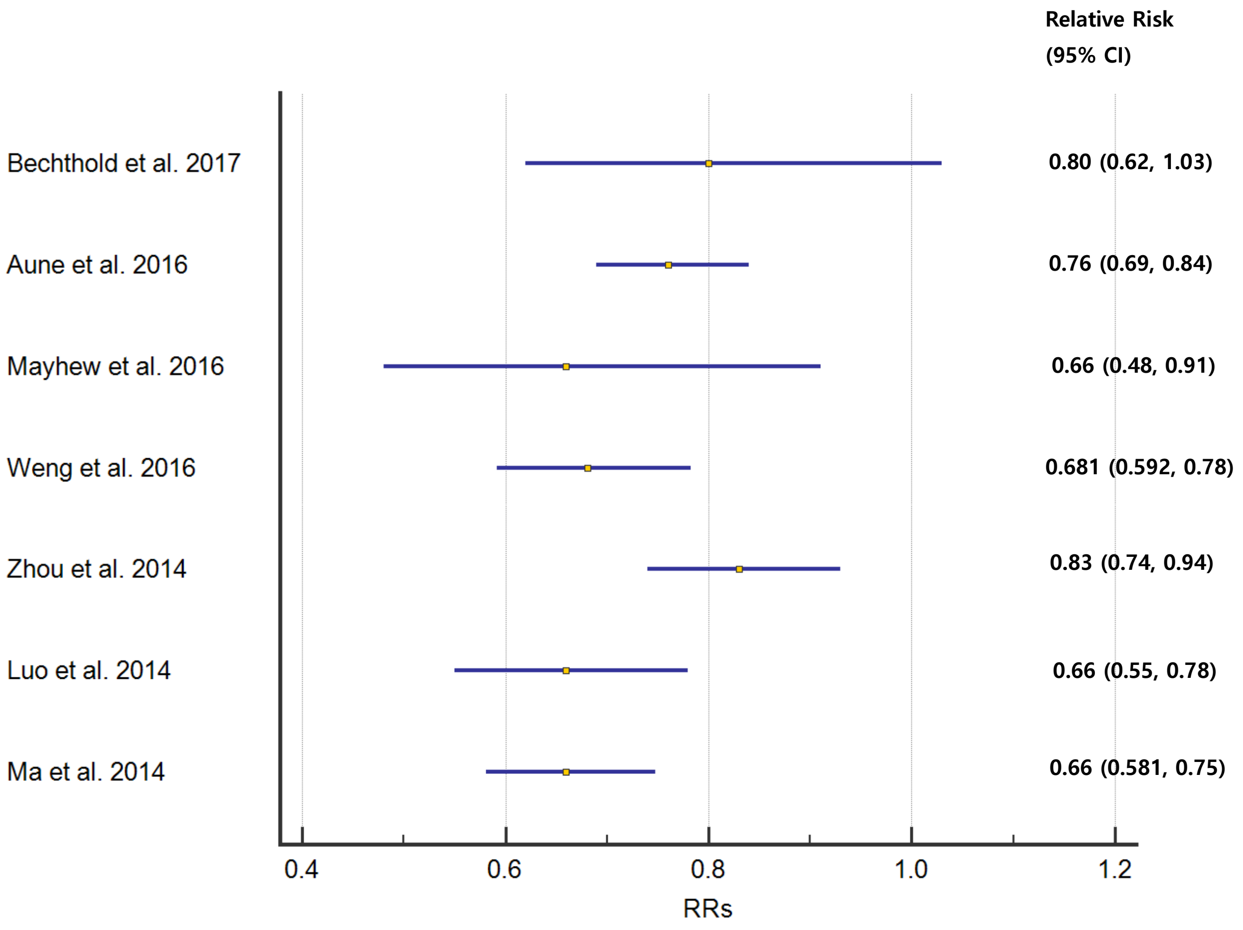

Supplementary Figure 18. Total nuts and total coronary heart disease, dose-response analysis

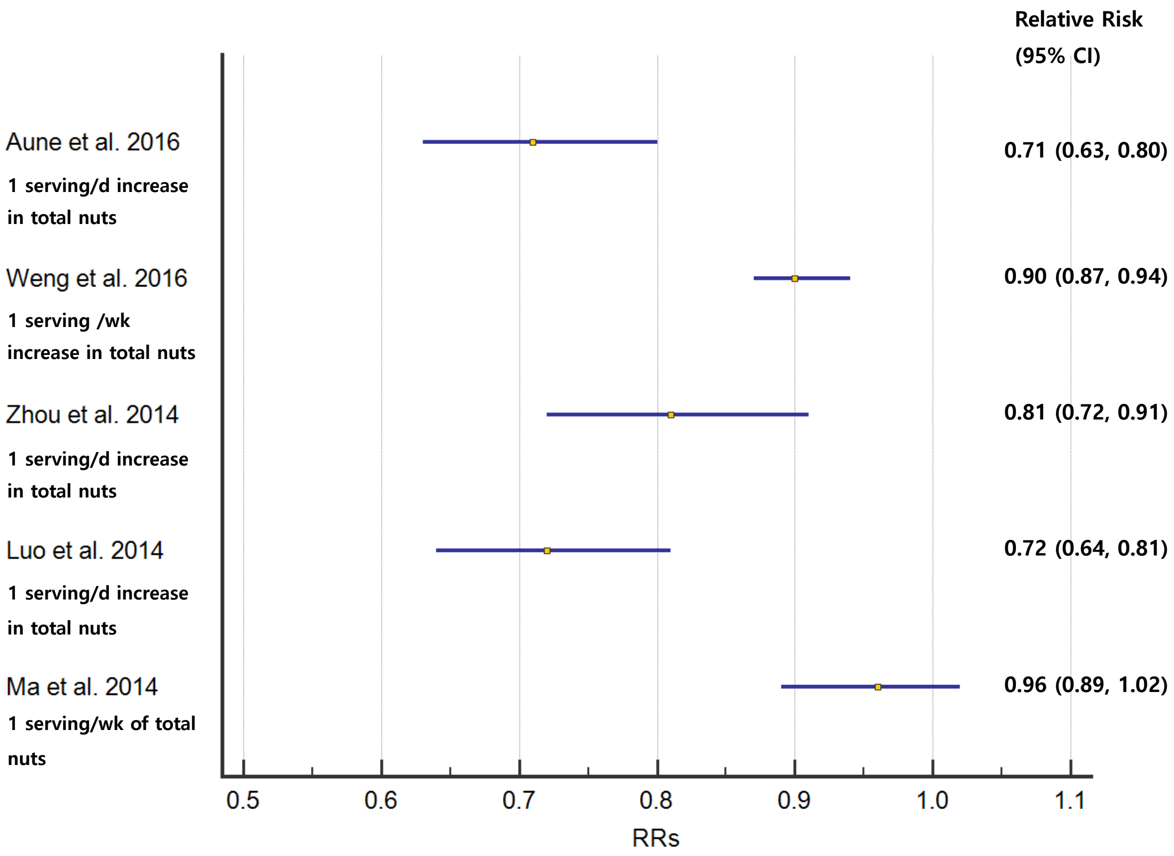

Supplementary Figure 19. Total nuts and non-fatal/fatal coronary heart disease, dose-response analysis

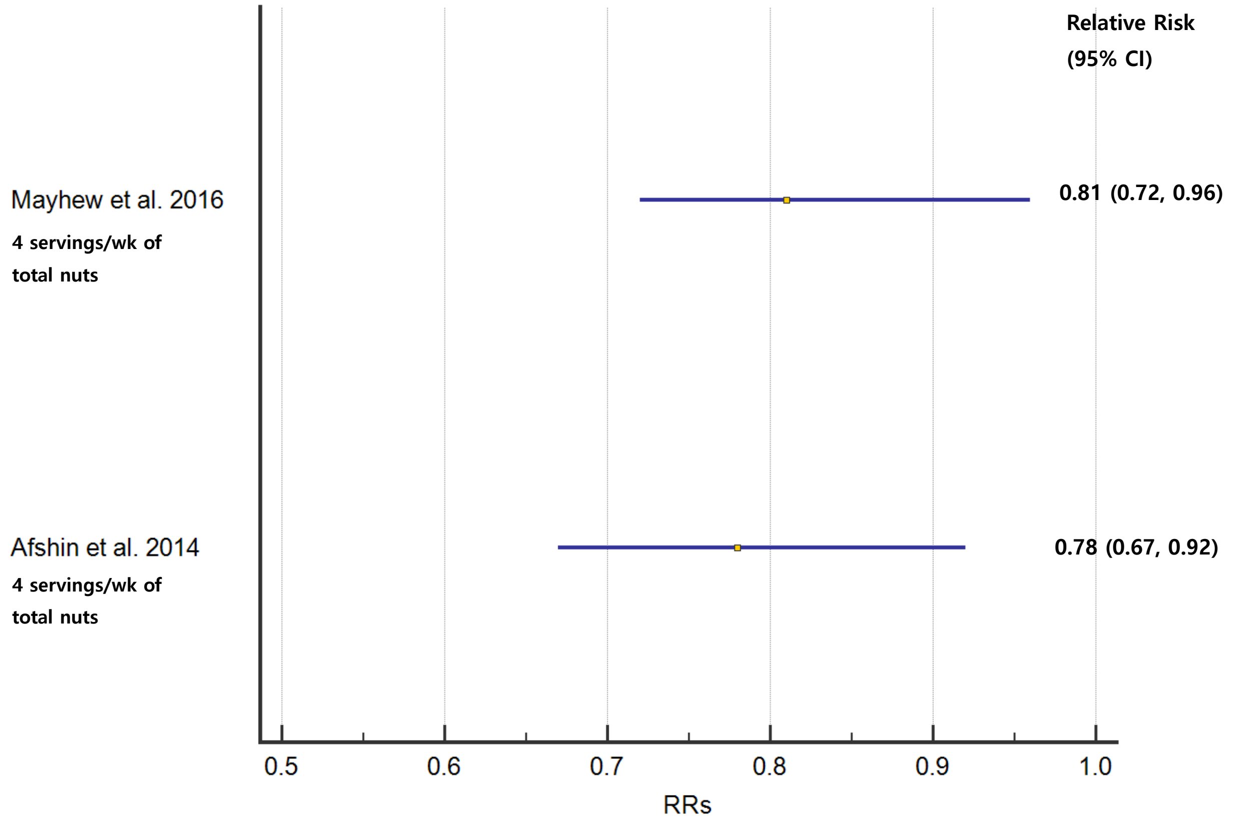

Supplementary Figure 20. Total nuts and total stroke, high *vs.* low analysis

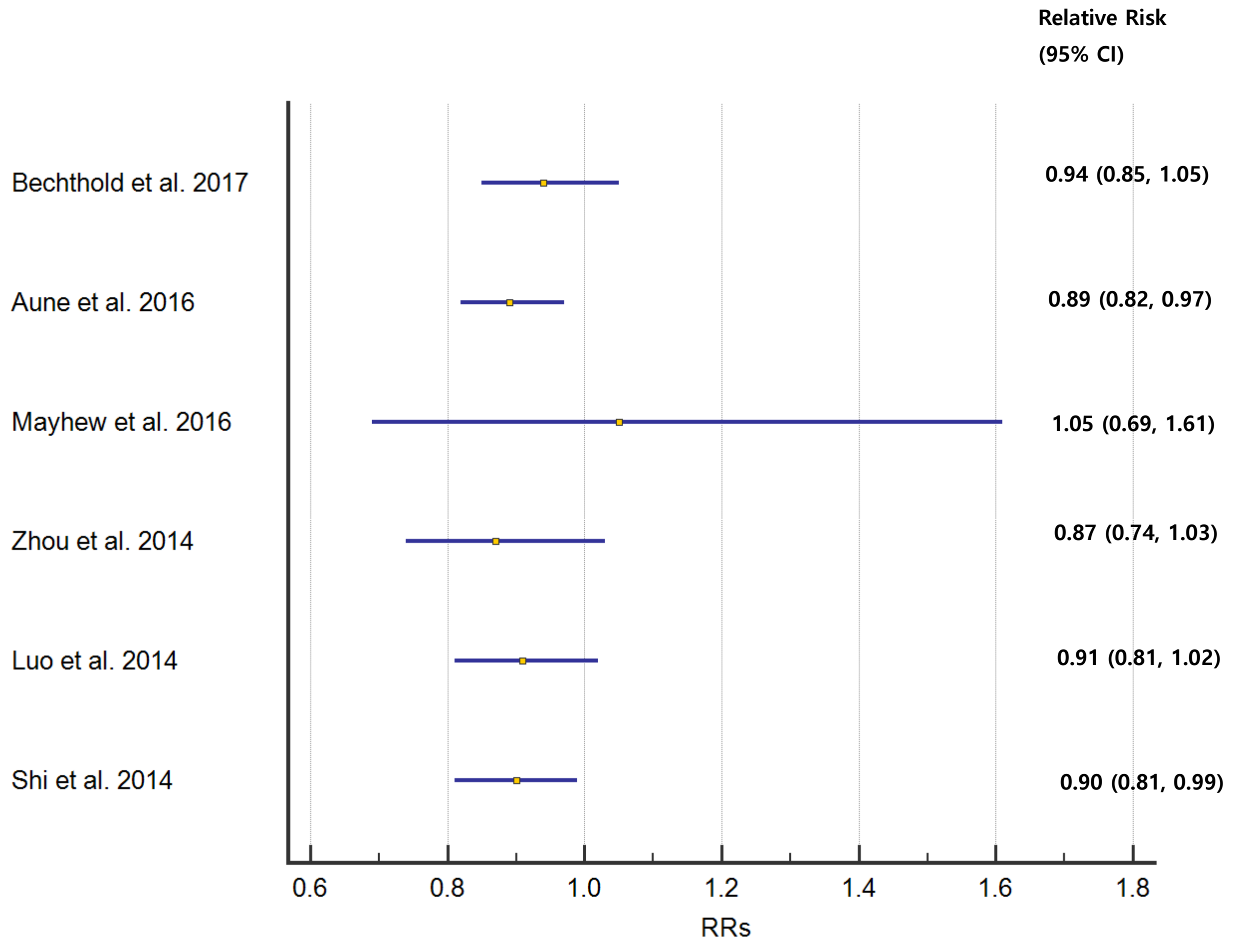

Supplementary Figure 21. Total nuts and total stroke, dose-response analysis

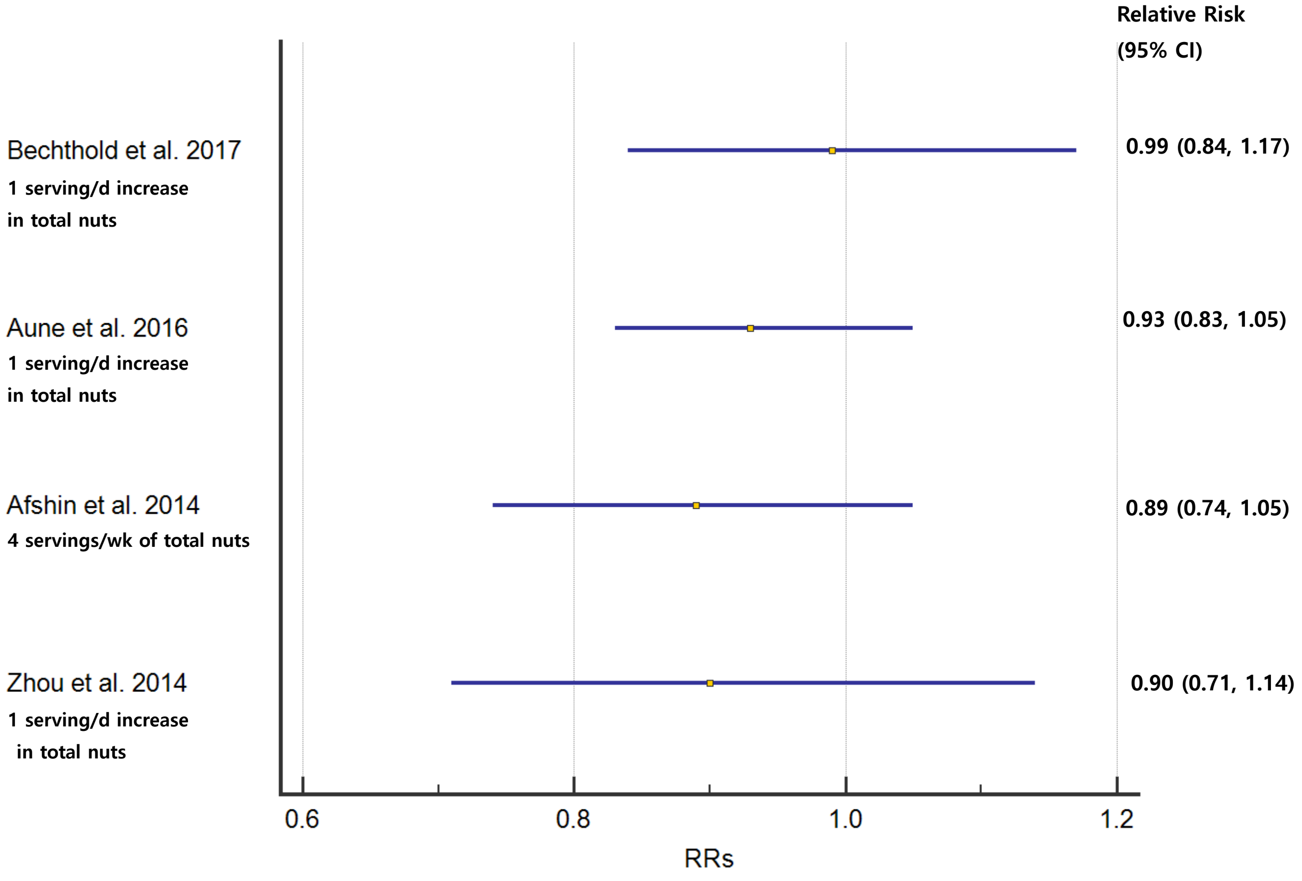

Supplementary Figure 22. Nuts and body weight

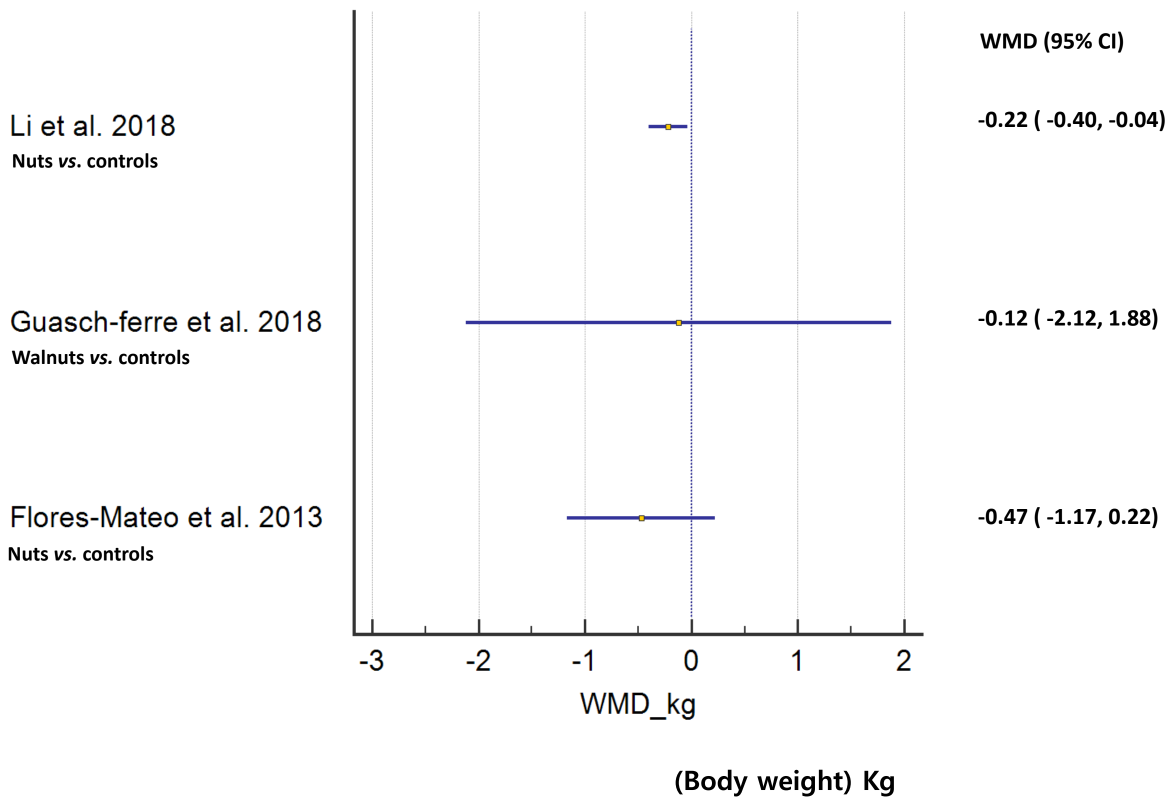

Supplementary Figure 23. Nuts and waist circumference

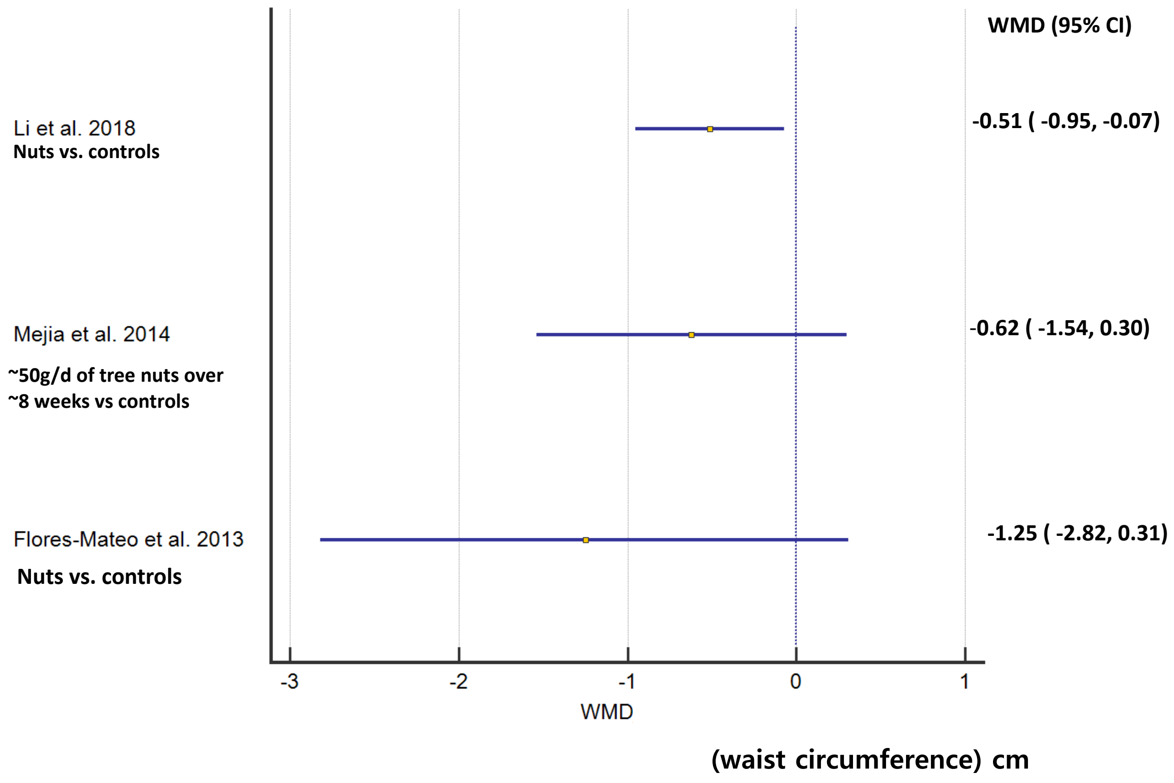

Supplementary Figure 24. Nuts and body mass index

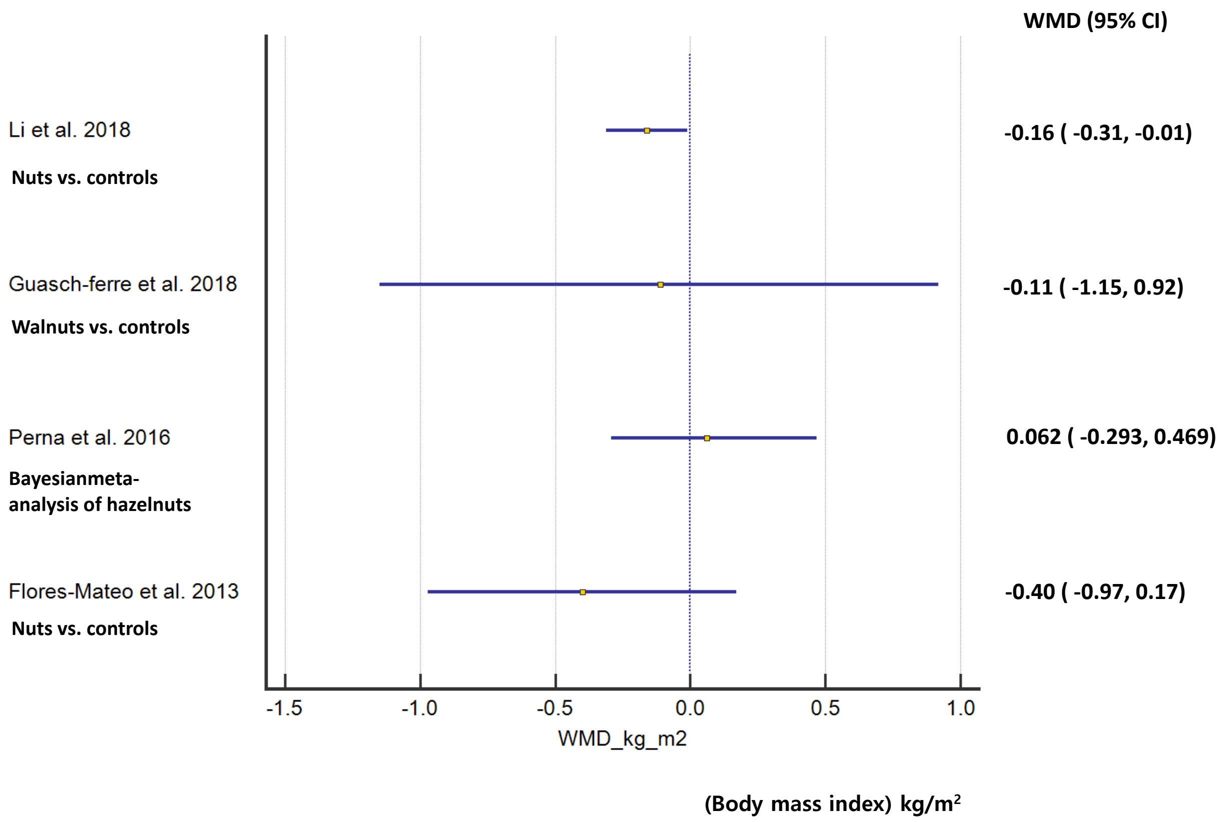

Supplementary Figure 25. Nuts and fasting blood glucose

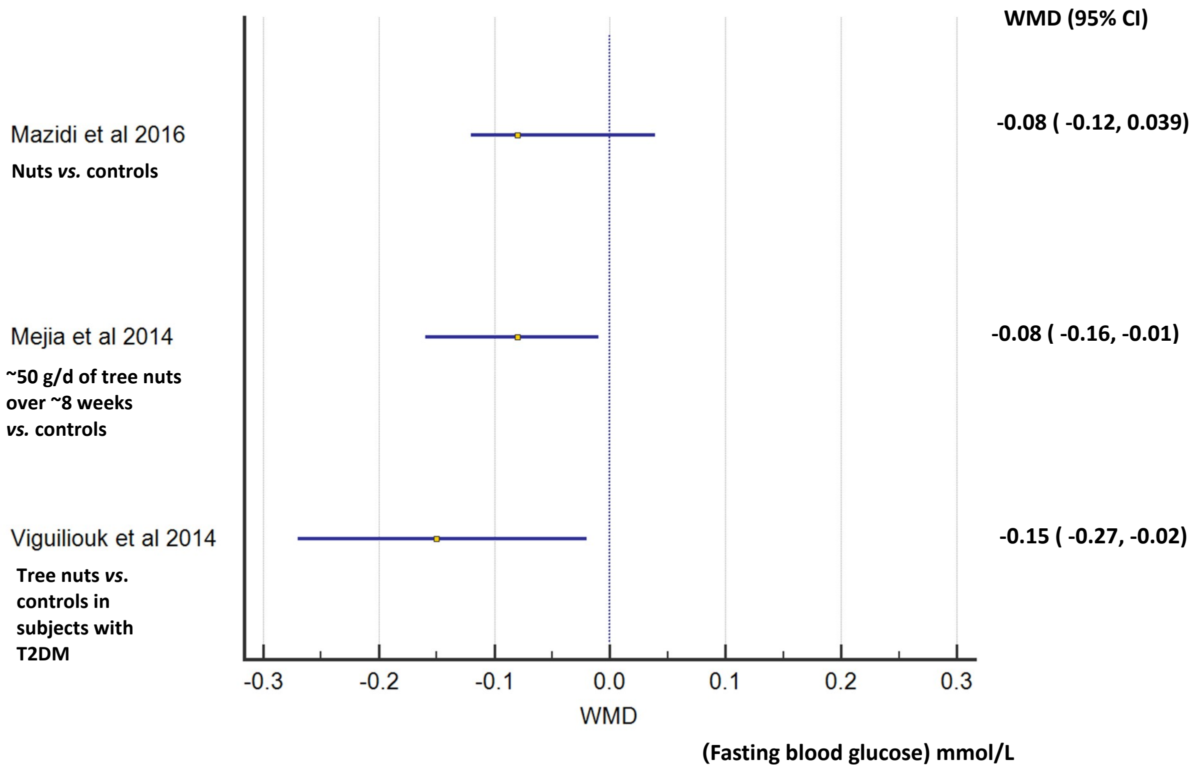

Supplementary Figure 26. Nuts and total cholesterol

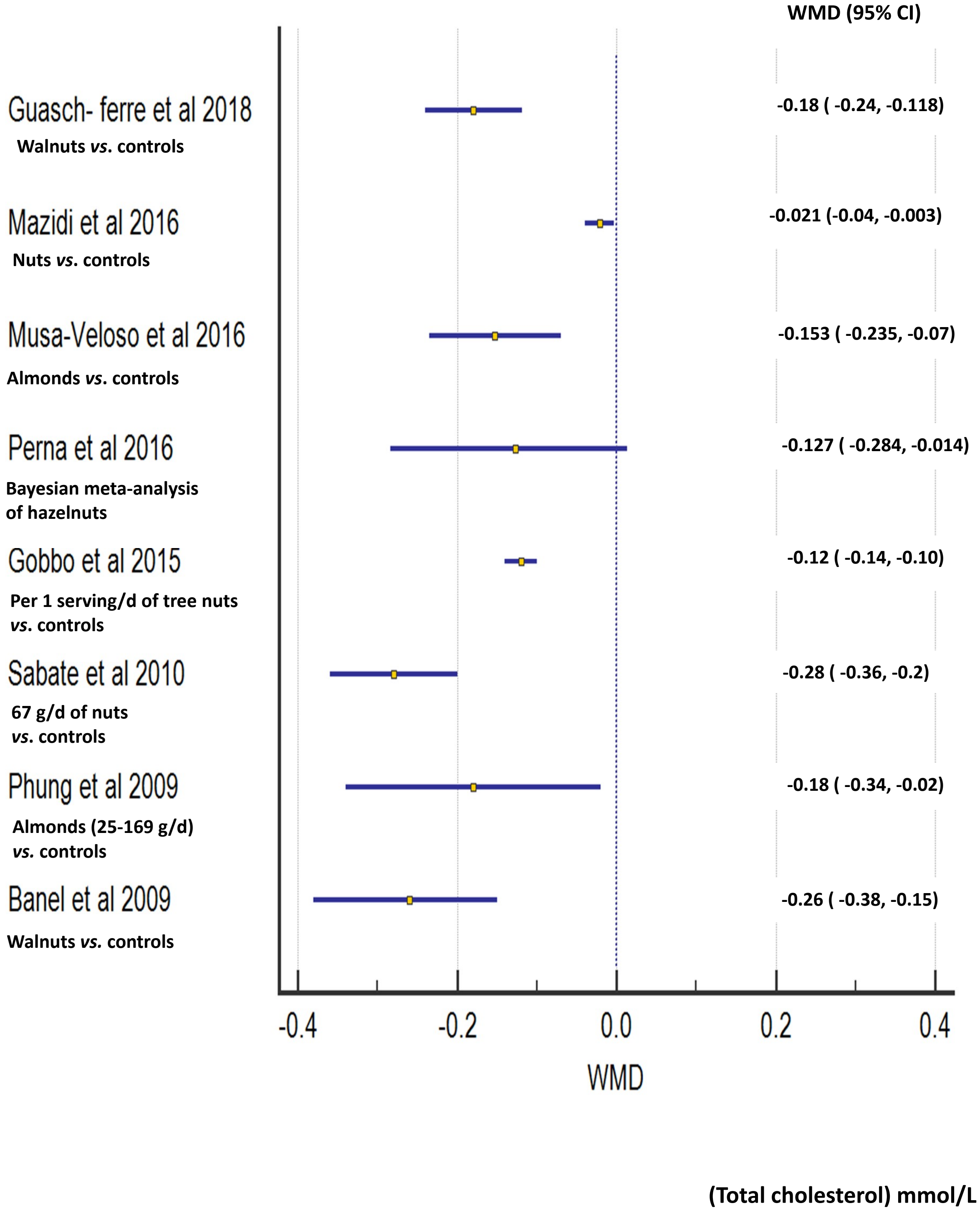

Supplementary Figure 27. Nuts and low-density lipoprotein cholesterol

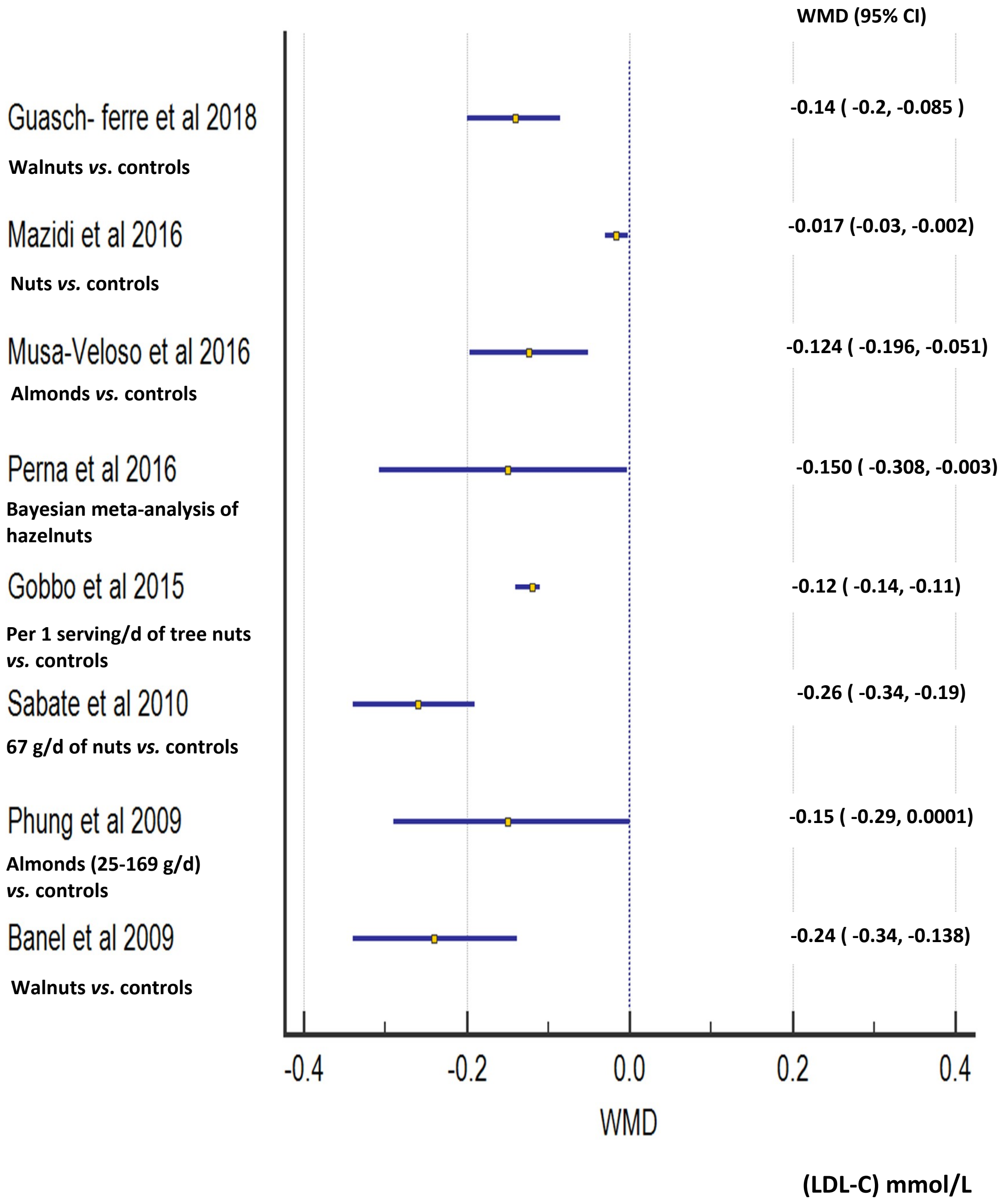

Supplementary Figure 28. Nuts and high-density lipoprotein cholesterol

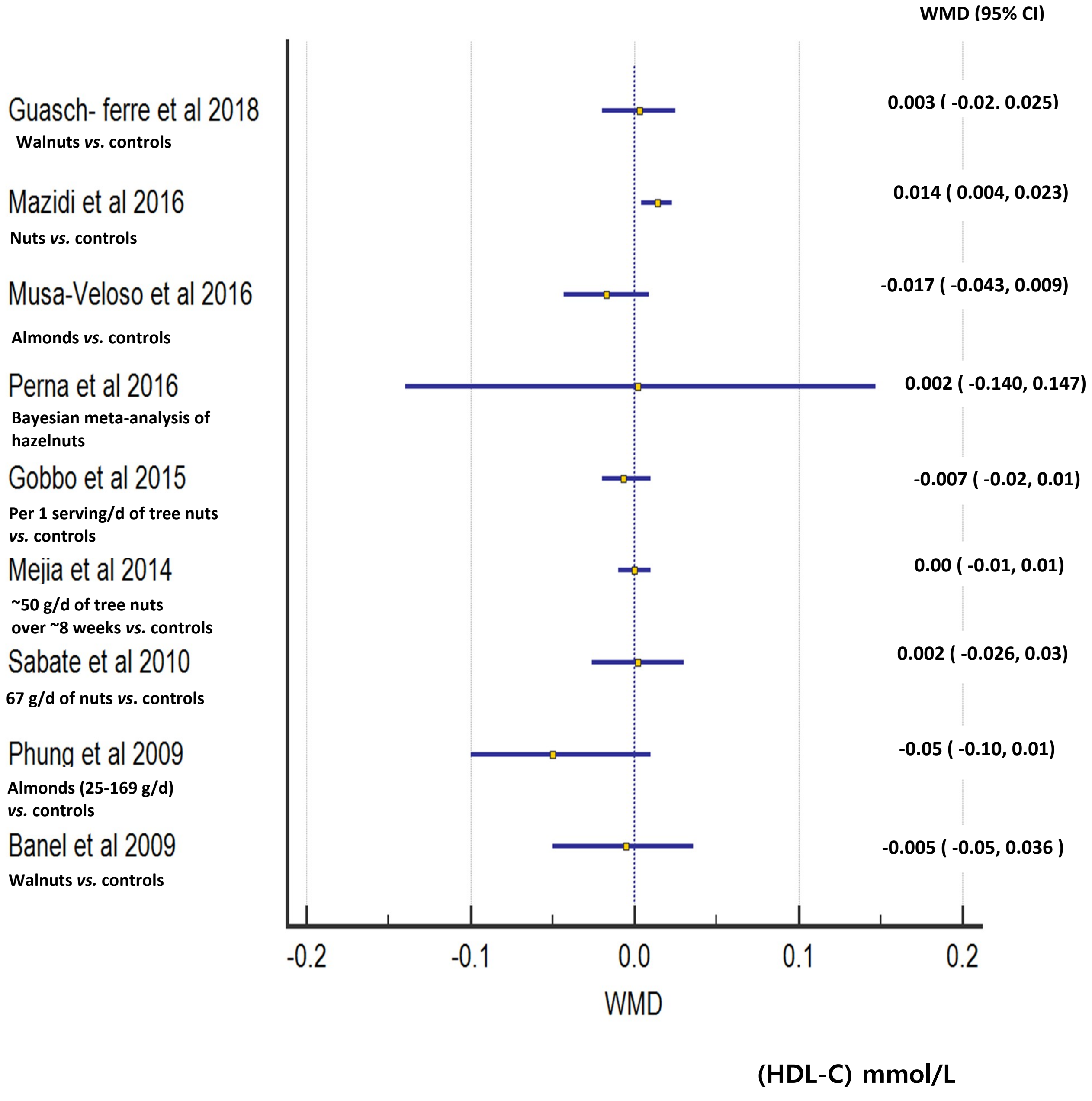

Supplementary Figure 29. Nuts and triglycerides

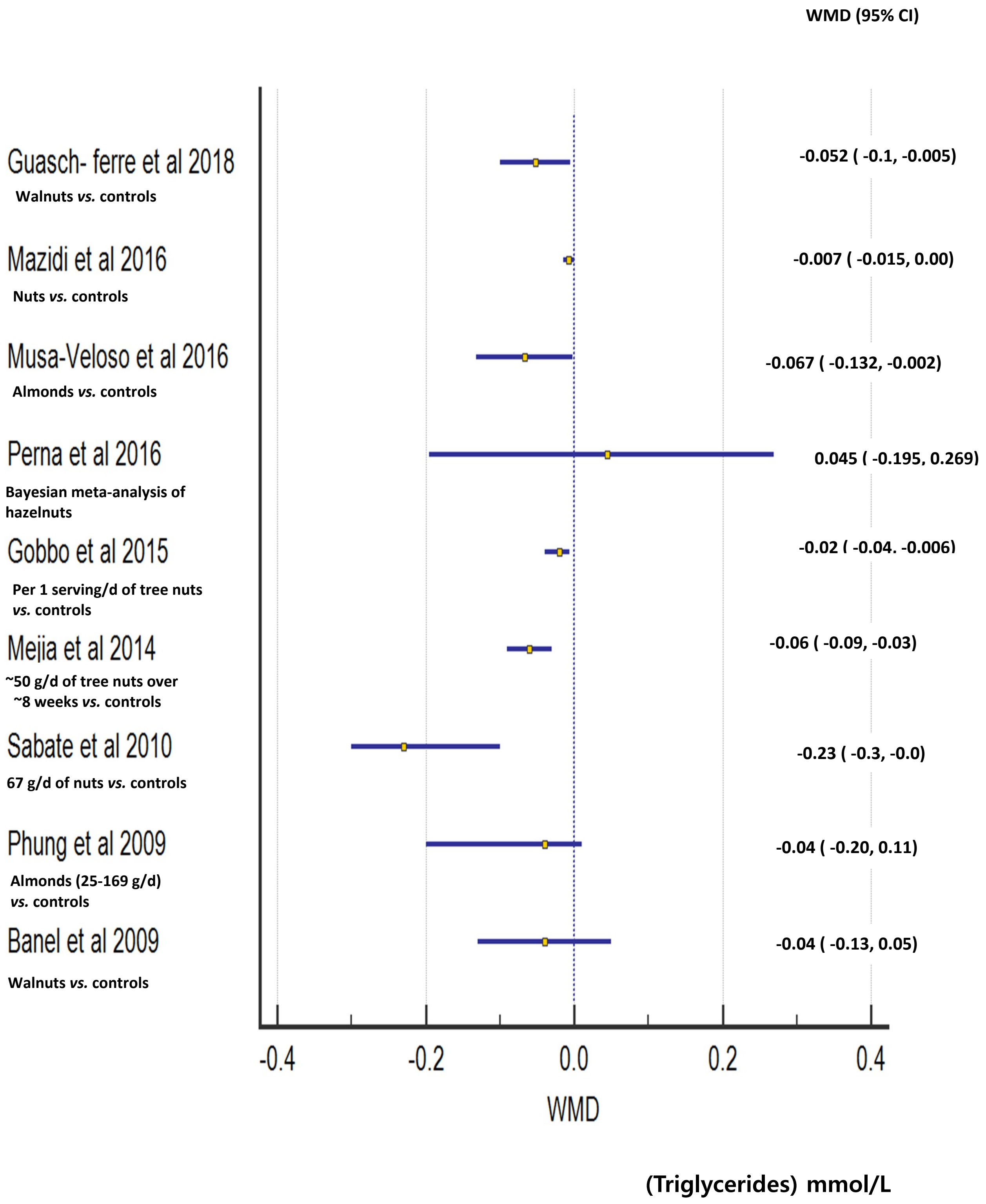

Supplementary Figure 30. Nuts and apolipoprotein A-1

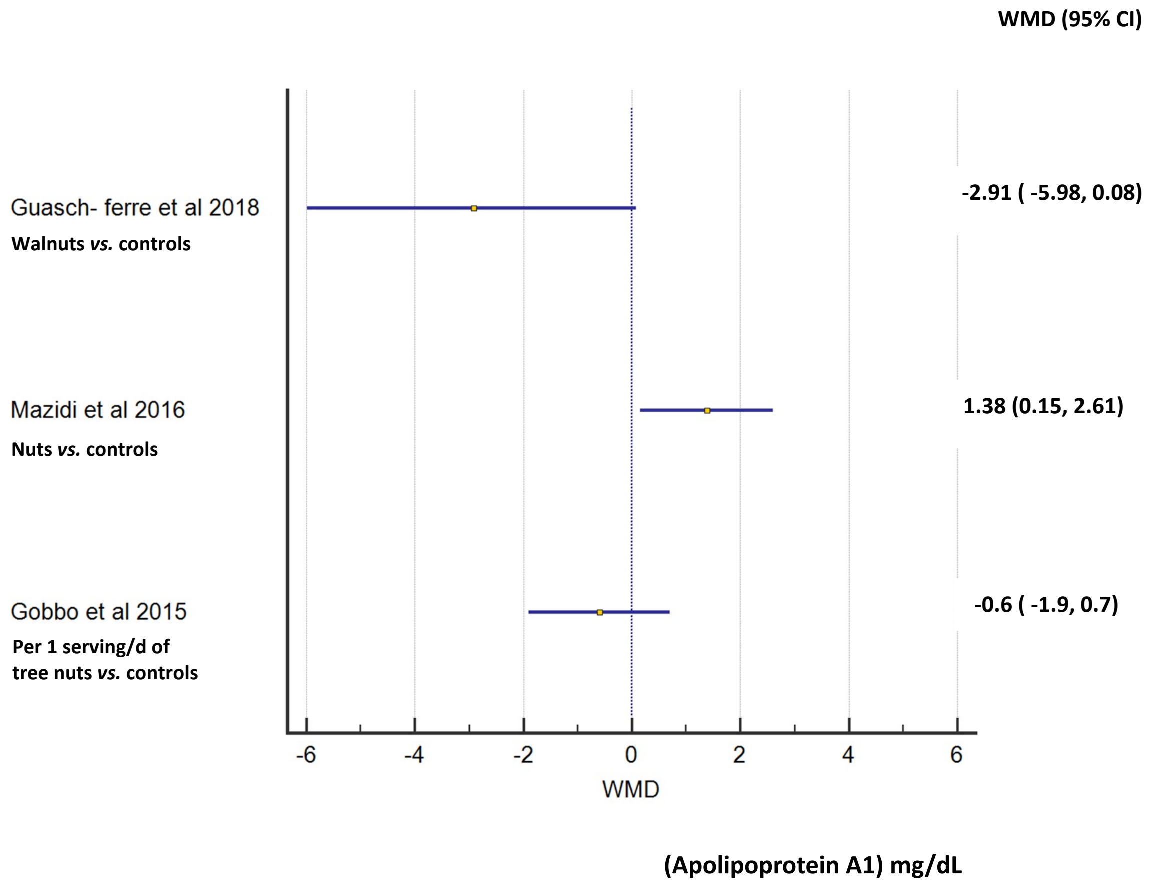

Supplementary Figure 31. Nuts and apolipoprotein B

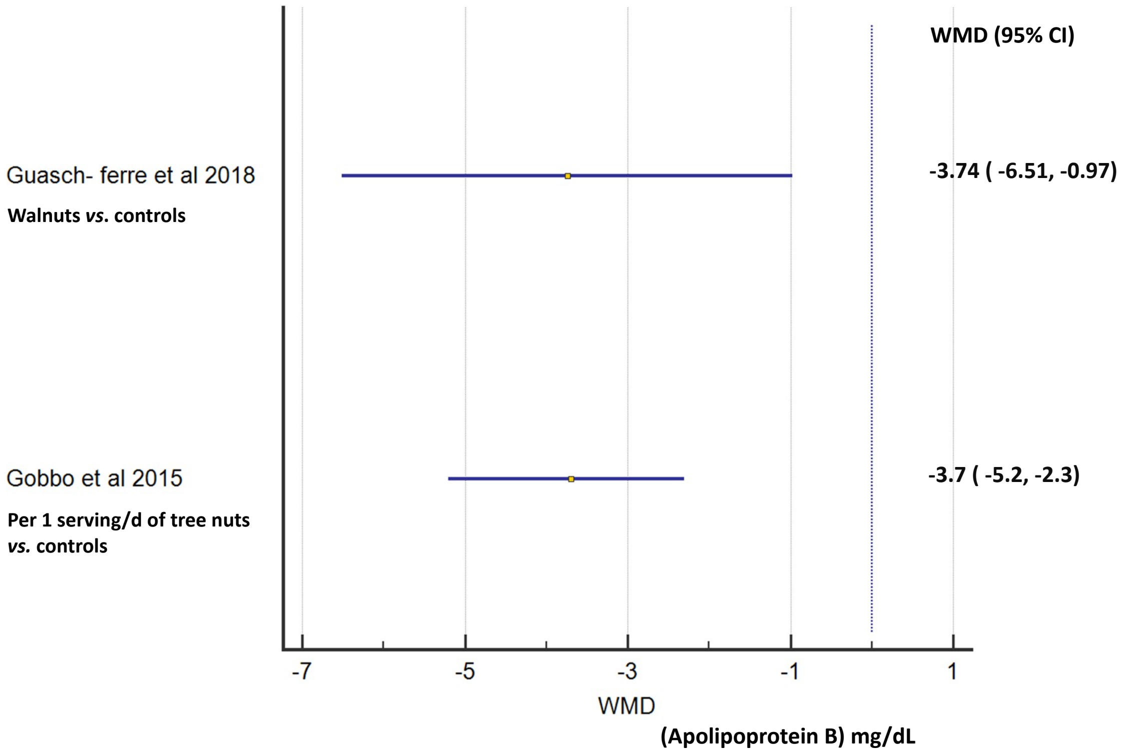

Supplementary Figure 32. Nuts and apolipoprotein B 100

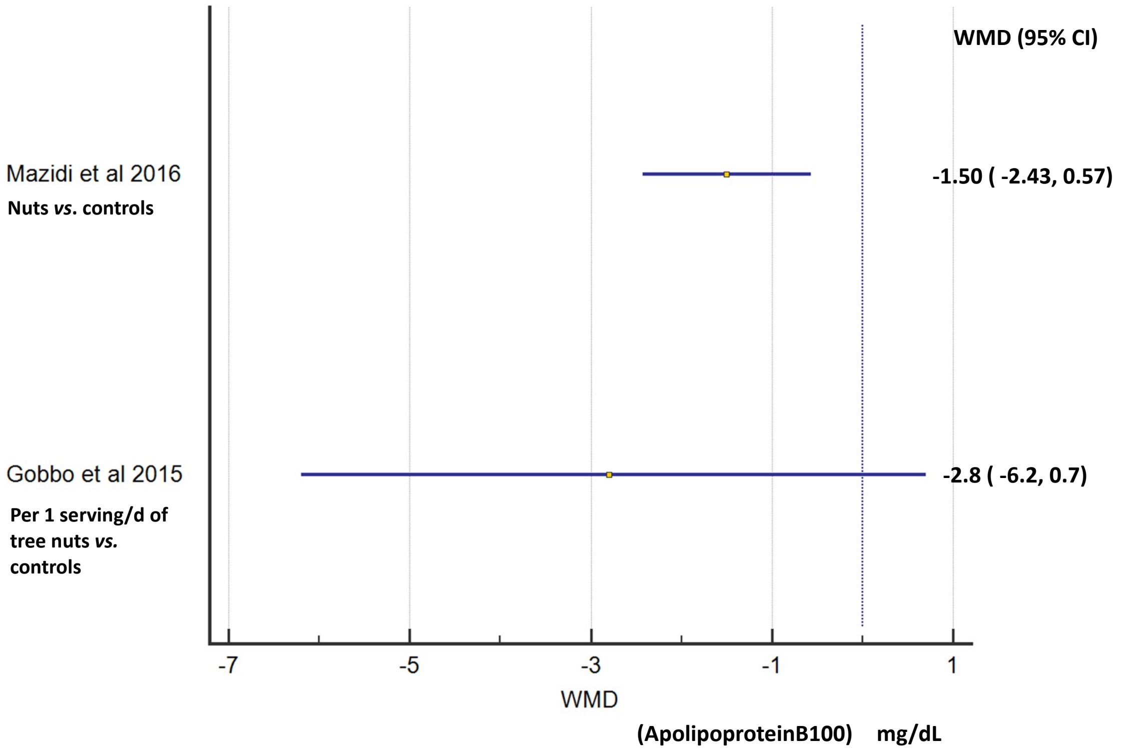

Supplementary Figure 33. Nuts and systolic blood pressure

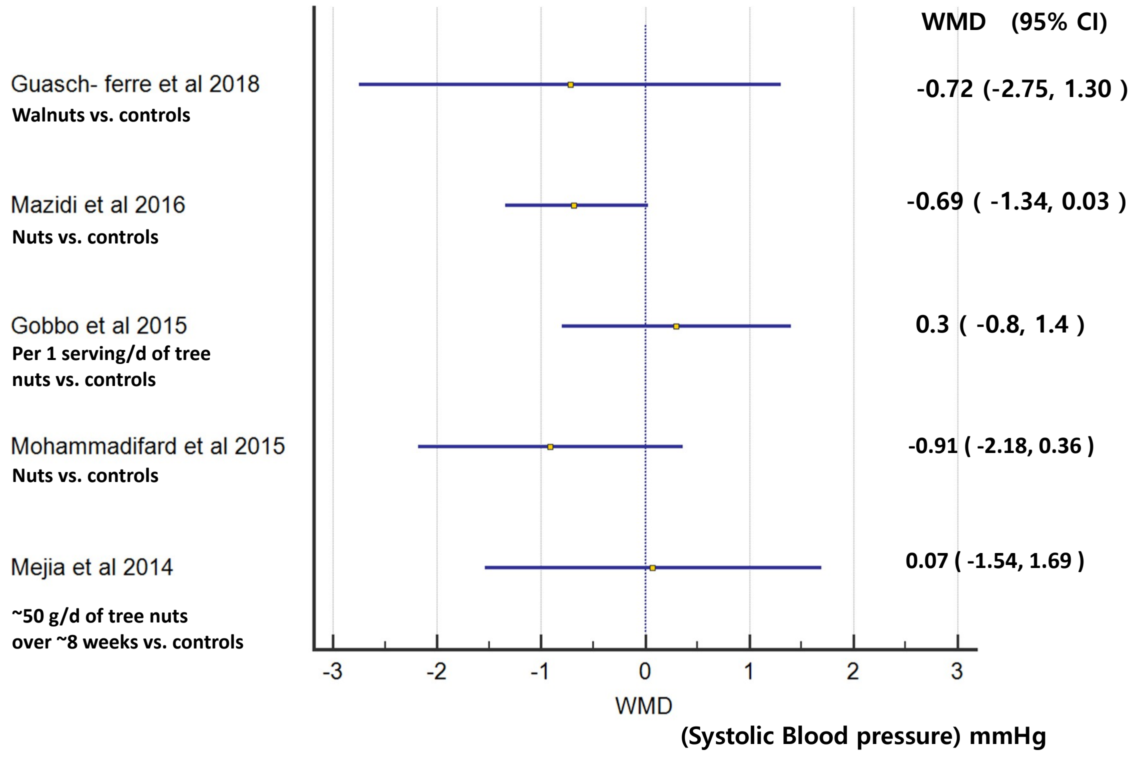

Supplementary Figure 34. Nuts and diastolic blood pressure

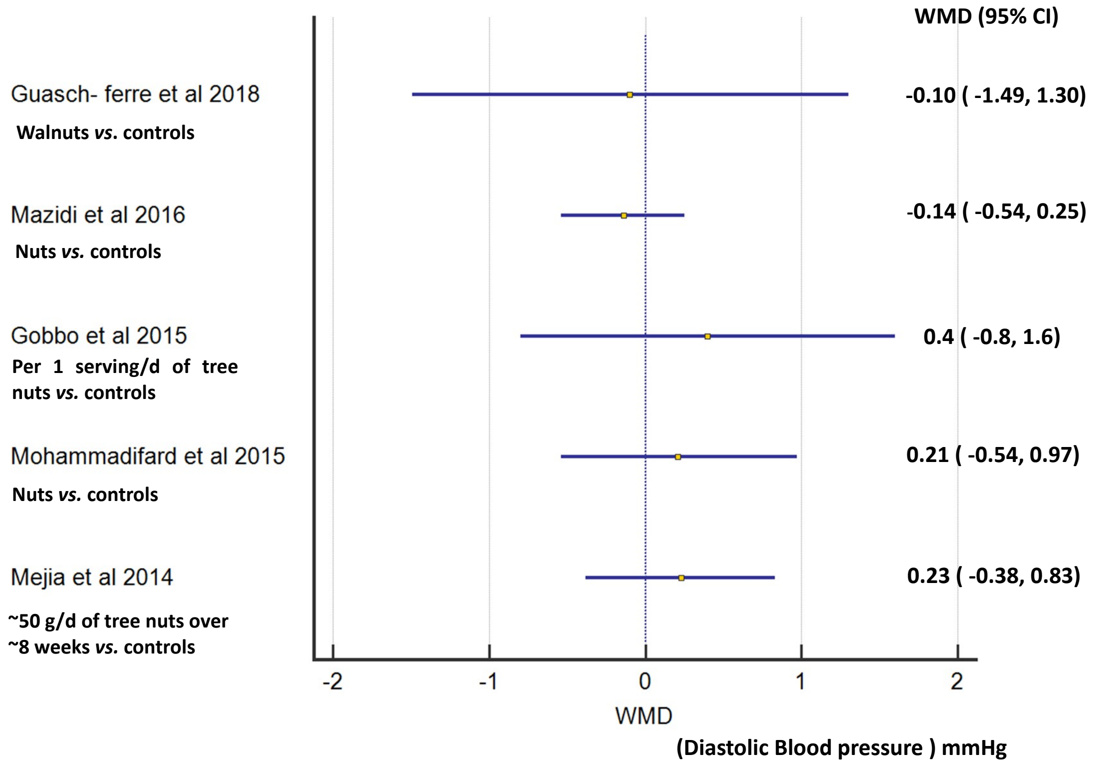

Supplementary Figure 35. Nuts and adiponectin

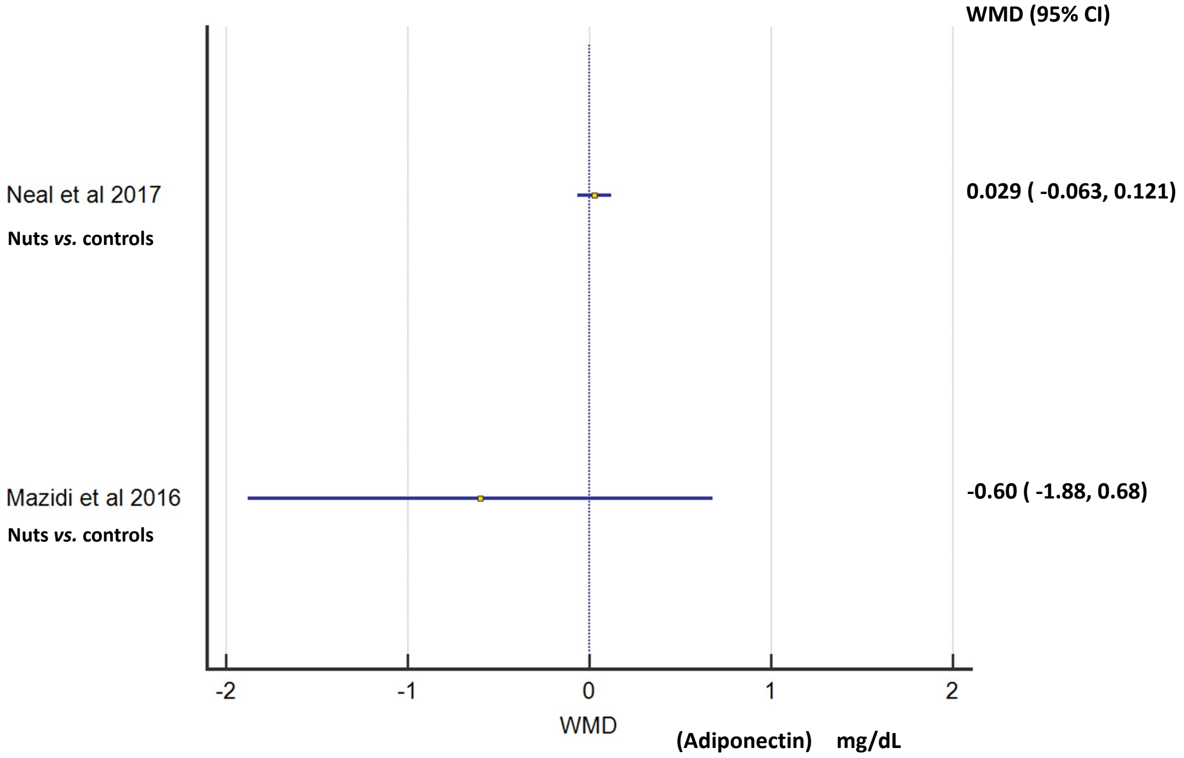

Supplementary Figure 36. Nuts and C-reactive protein (CRP)

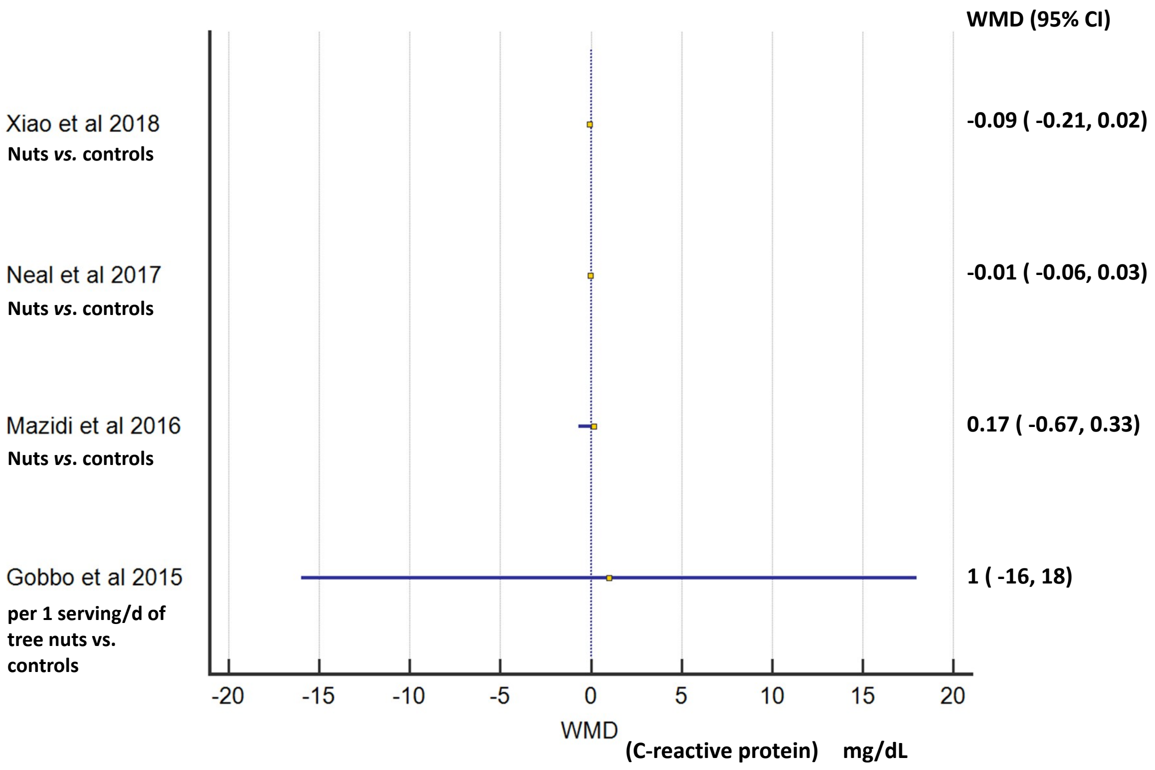

Supplementary Figure 37. Nuts and interleukin-6 (IL-6)

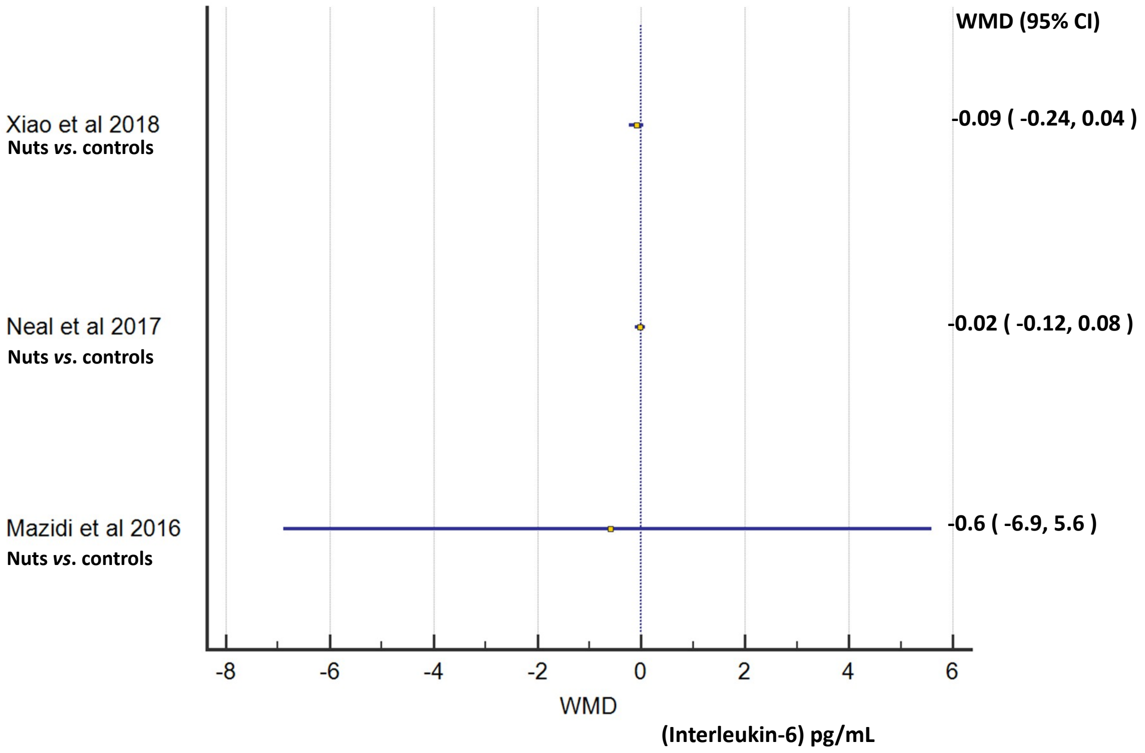

Supplementary Figure 38. Nuts and tumor necrosis factor-alpha (TNF-α)

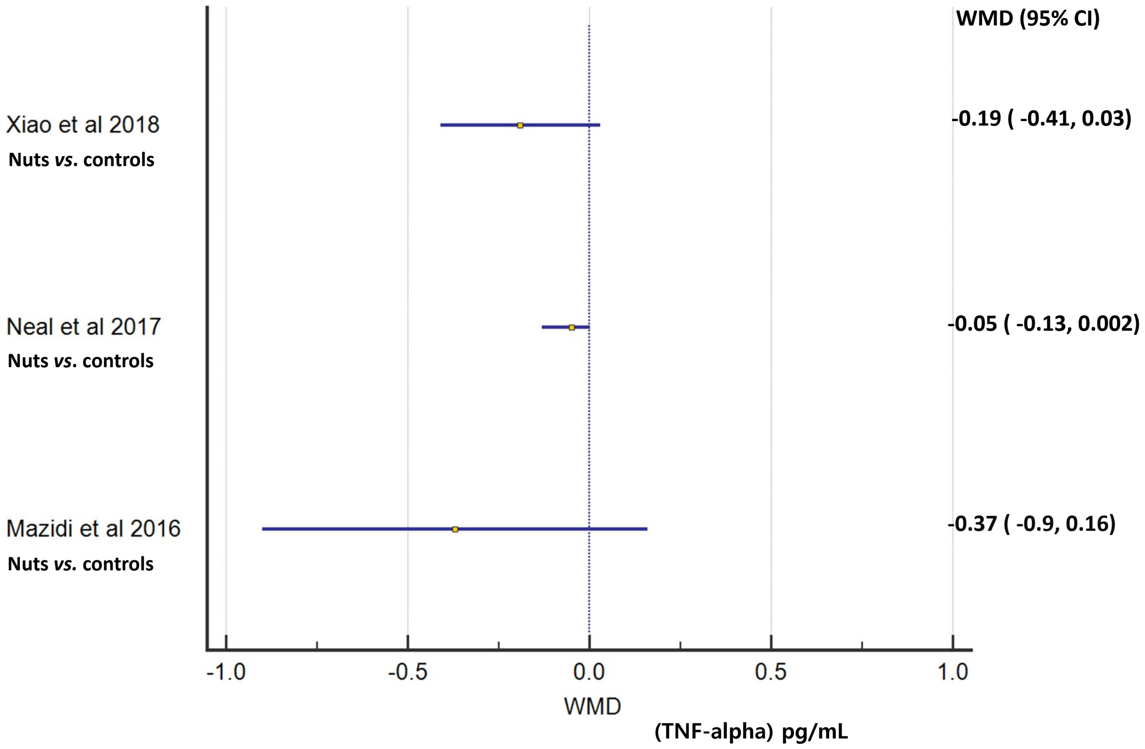

Supplementary Figure 39. Nuts and intracellular adhesion molecule-1 (ICAM-1)

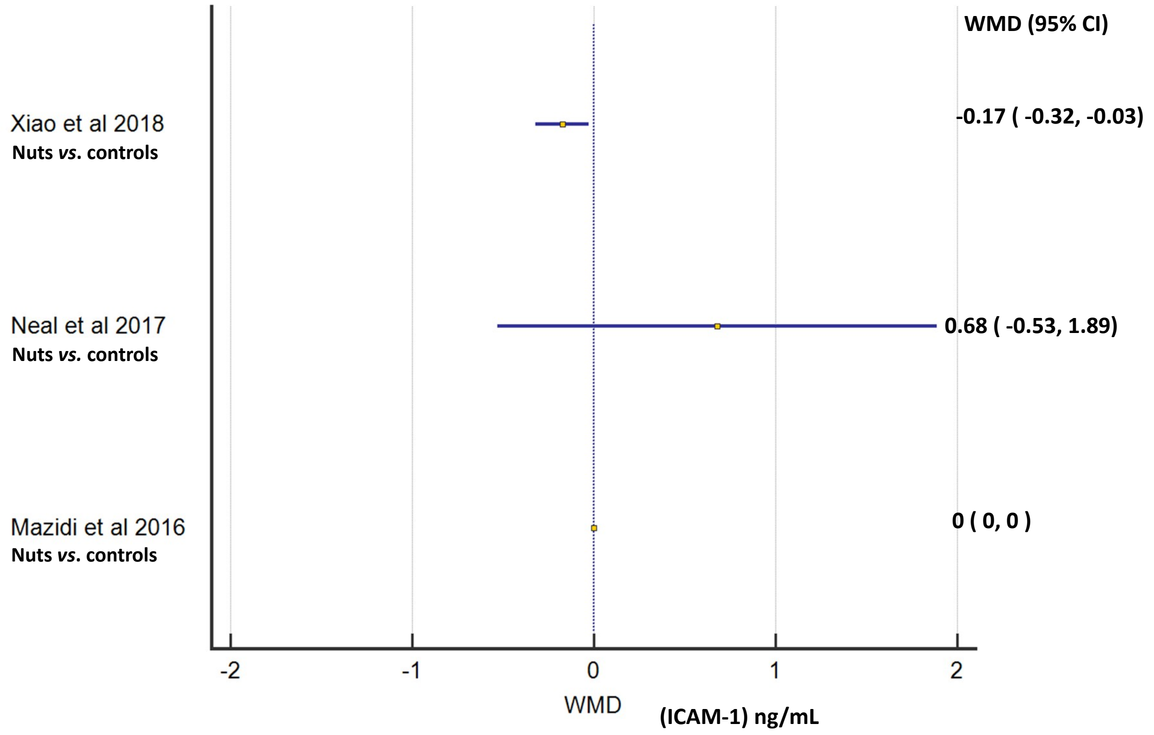

Supplementary Figure 40. Nuts and vascular cell adhesion molecule-1 (VCAM-1)

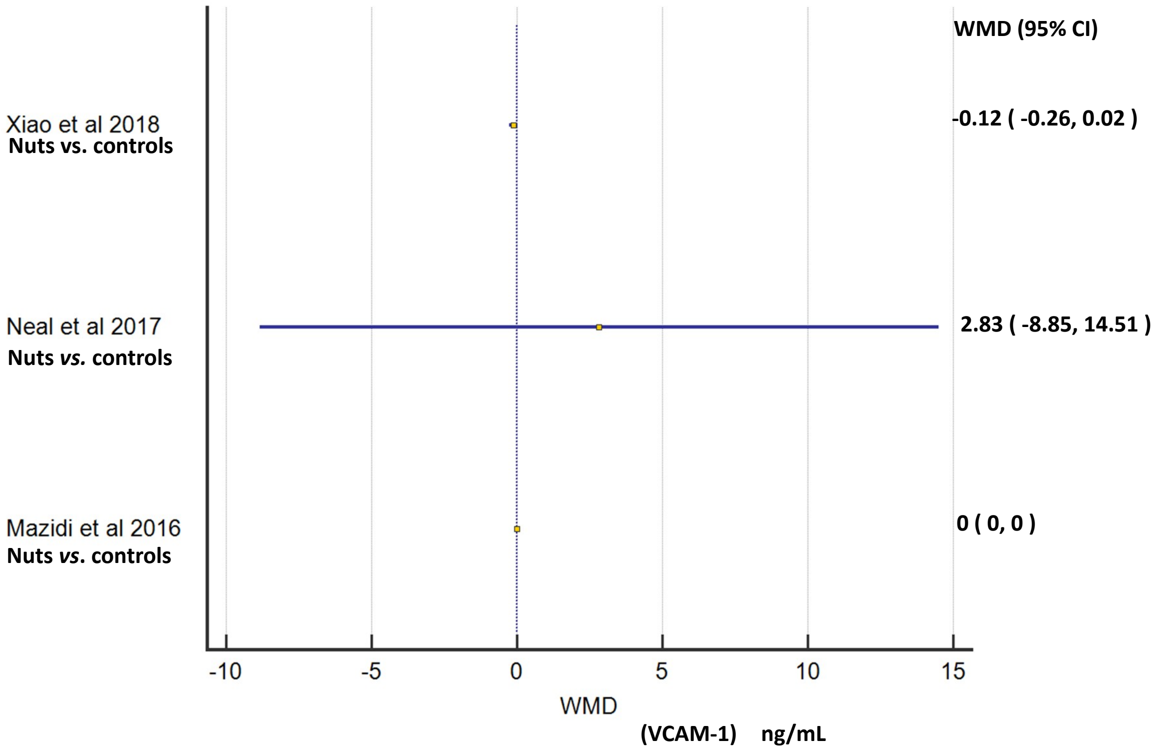

Supplementary Figure 41. Nuts and E-selectin

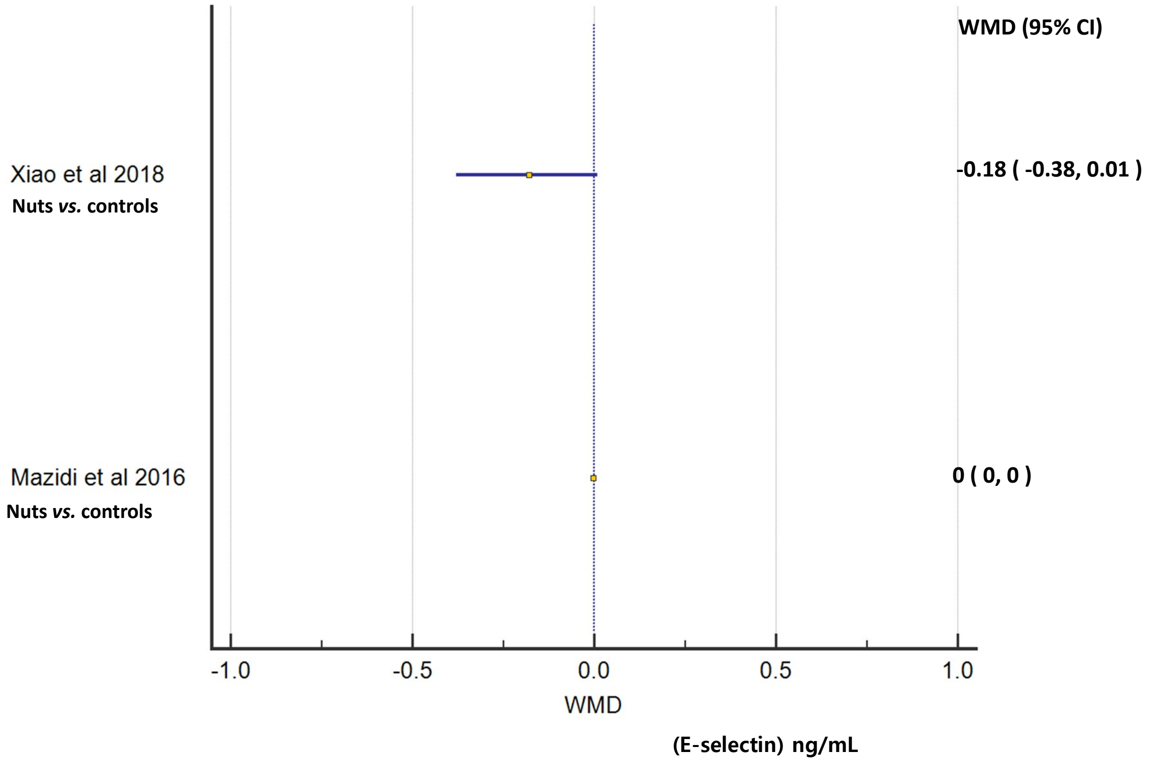

Supplementary Figure 42. Nuts and flow-mediated dilation (FMD)

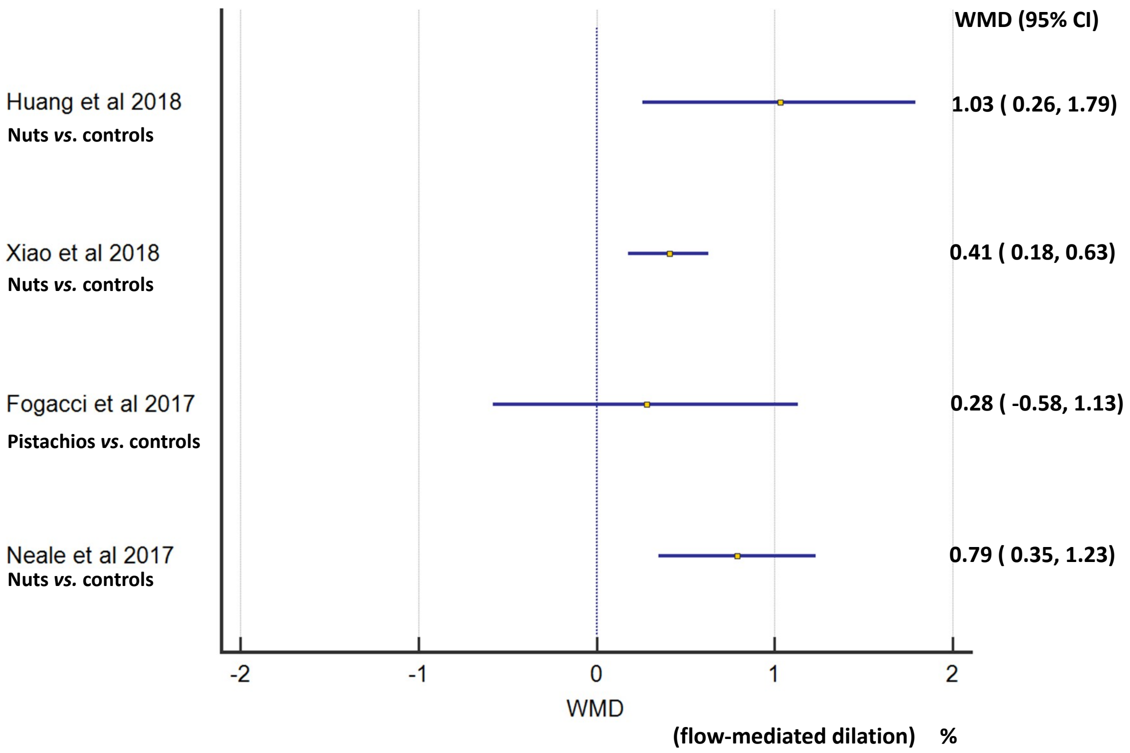

Supplement: Supplementary file 1 [file nutrients-10-01935-s001.pdf]
